# Evidence of SARS-CoV-2 infection in postmortem lung, kidney, and liver samples, revealing cellular targets involved in COVID-19 pathogenesis

**DOI:** 10.1007/s00705-023-05711-y

**Published:** 2023-02-26

**Authors:** Viviana Falcón-Cama, Teresita Montero-González, Emilio F. Acosta-Medina, Gerardo Guillen-Nieto, Jorge Berlanga-Acosta, Celia Fernández-Ortega, Anabel Alfonso-Falcón, Nathalie Gilva-Rodríguez, Lilianne López-Nocedo, Daina Cremata-García, Mariuska Matos-Terrero, Giselle Pentón-Rol, Iris Valdés, Leonardo Oramas-Díaz, Anamarys Suarez-Batista, Enrique Noa-Romero, Otto Cruz-Sui, Daisy Sánchez, Amanda I. Borrego-Díaz, Juan E. Valdés-Carreras, Ananayla Vizcaino, José Suárez-Alba, Rodolfo Valdés-Véliz, Gretchen Bergado, Miguel A. González, Tays Hernandez, Rydell Alvarez-Arzola, Anna C. Ramírez-Suárez, Dionne Casillas-Casanova, Gilda Lemos-Pérez, Omar R. Blanco-Águila, Angelina Díaz, Yorexis González, Mónica Bequet-Romero, Javier Marín-Prida, Julio C. Hernández-Perera, Leticia del Rosario-Cruz, Alina P. Marin-Díaz, Maritza González-Bravo, Israel Borrajero, Nelson Acosta-Rivero

**Affiliations:** 1 Present Address: Center for Genetic Engineering and Biotechnology (CIGB), Ave 31 be/ 158 and 190, Cubanacán, Playa, PO Box 6162, 10699 Havana, Cuba; 2 Hospital “Luis Díaz Soto”, Havana, Cuba; 3 Center for Advanced Studies of Cuba, Havana, Cuba; 4 Hospital and Center for Clinic and Chirurgic Research, Havana, Cuba; 5 Department of Virology, Civilian Defense Scientific Research Center (CICDC), Havana, Mayabeque Cuba; 6 Direction of Immunology and Immunotherapy, Center of Molecular Immunology, Havana, Cuba; 7 CIGB, Sancti Spíritus, Cuba; 8 Center for Research and Biological Evaluations, Institute of Pharmacy and Food, University of Havana, Havana, Cuba; 9 International Orthopedic Scientific Complex ‘Frank Pais Garcia’, Havana, Cuba; 10 Latin American School of Medicine, Calle Panamericana Km 3 1/2, Playa, 11600 Havana, Cuba; 11 Clinical Surgical Hospital ‘Hermanos Ameijeiras’, Havana, Cuba; 12 Center for Protein Studies, Department of Biochemistry, Faculty of Biology, University of Habana, Calle 25 entre J e I, #455, Plaza de la Revolucion, 10400 Havana, Cuba; 13 Present Address: Department of Infectious Diseases, Centre for Integrative Infectious Disease Research (CIID), Molecular Virology, University of Heidelberg, Medical Faculty Heidelberg, INF 344, GO.1, 69120 Heidelberg, Germany

## Abstract

There is an urgent need to understand severe acute respiratory syndrome coronavirus 2 (SARS-CoV-2)-host interactions involved in virus spread and pathogenesis, which might contribute to the identification of new therapeutic targets. In this study, we investigated the presence of SARS-CoV-2 in postmortem lung, kidney, and liver samples of patients who died with coronavirus disease (COVID-19) and its relationship with host factors involved in virus spread and pathogenesis, using microscopy-based methods. The cases analyzed showed advanced stages of diffuse acute alveolar damage and fibrosis. We identified the SARS-CoV-2 nucleocapsid (NC) in a variety of cells, colocalizing with mitochondrial proteins, lipid droplets (LDs), and key host proteins that have been implicated in inflammation, tissue repair, and the SARS-CoV-2 life cycle (vimentin, NLRP3, fibronectin, LC3B, DDX3X, and PPARγ), pointing to vimentin and LDs as platforms involved not only in the viral life cycle but also in inflammation and pathogenesis. SARS-CoV-2 isolated from a patient´s nasal swab was grown in cell culture and used to infect hamsters. Target cells identified in human tissue samples included lung epithelial and endothelial cells; lipogenic fibroblast-like cells (FLCs) showing features of lipofibroblasts such as activated PPARγ signaling and LDs; lung FLCs expressing fibronectin and vimentin and macrophages, both with evidence of NLRP3- and IL1β-induced responses; regulatory cells expressing immune-checkpoint proteins involved in lung repair responses and contributing to inflammatory responses in the lung; CD34^+^ liver endothelial cells and hepatocytes expressing vimentin; renal interstitial cells; and the juxtaglomerular apparatus. This suggests that SARS-CoV-2 may directly interfere with critical lung, renal, and liver functions involved in COVID-19-pathogenesis.

**Supplementary Information:**

The online version contains supplementary material available at 10.1007/s00705-023-05711-y.

## Introduction

Infection with severe acute respiratory syndrome coronavirus 2 (SARS-CoV-2) leading to coronavirus disease 19 (COVID-19) has been posing a great threat to global public health since 2020 [111, 121]. There is an urgent need to understand virus-host interactions involved in the mechanisms of SARS-CoV-2 infection and pathogenesis that may contribute to the identification of new therapeutic targets. About 20% of COVID-19 patients develop serious manifestations such as severe pneumonia, acute respiratory distress syndrome (ARDS), sepsis, and death [104].

SARS-CoV-2 is an enveloped positive-sense single-stranded RNA virus with a genome size of approximately 30 kb [100]. Two overlapping open reading frames (ORFs) are translated from the 5’ region: ORF1a and ORF1b. The latter is translated from a -1 frameshift that allows a large polyprotein to be produced beyond the stop codon of ORF1a. The two polyproteins are proteolytically processed by viral proteases to yield the non-structural proteins NSP1-NSP16. Additional smaller ORFs encode the structural proteins: spike (S), envelope (E), membrane (M), nucleocapsid (NC), and other polypeptides [30]. Angiotensin-converting enzyme (ACE) 2 (ACE2) has been identified as the main functional receptor of SARS-CoV-2, interacting with the viral S protein. Importantly, the primary physiological role of ACE2 is the regulation of vasoconstriction and blood pressure [23].

Detection of SARS-CoV-2 in different organs and various COVID-19 manifestations such as cardiovascular and nervous system complications, kidney injury, and gastrointestinal tract symptoms suggest that extrapulmonary sites of infection contribute to disease pathogenesis [8, 11, 13, 25, 68, 70, 73, 90]. In particular, the kidney has been shown to be involved in COVID-19 pathogenesis, and renal injury is associated with morbidity and mortality [55]. Postmortem analysis and the possible impact of SARS-CoV-2 on different organs are valuable for understanding virus spread and the pathophysiological mechanisms of infection. Investigating the cell tropism of the virus and its role in virus-induced pathogenesis is especially important for understanding the mechanisms of SARS-CoV-2 infection and identifying new therapeutic targets. In this work, we investigated the presence of SARS-CoV-2 in various tissues of patients who died with COVID-19 and its relationship with host factors involved virus-induced pathogenesis. We identified potential cellular and molecular targets that may be involved in and affected by SARS-CoV-2 infection, with implications for virus-induced pathogenesis and therapeutics.

## Materials and methods

### Patients

Five patients (R, J, D, B, and T) with a nasopharyngeal swab that was positive for SARS-CoV-2 by real-time RT-PCR (qRT-PCR) [67] and who died with COVID-19 from April to September 2020, were studied in this work. Lung samples were also obtained from a person who died from a cause that was unrelated to COVID-19 (Table 1). These cases were part of a larger cohort whose main pathological findings have been summarized previously [15].

**Table 1:** Features of patients and clinical presentation

Patient	Age/sex	Symptoms	Coexisting conditions	Chest radiograph; treatment	Altered blood laboratory findings	Time of death from onset of symptoms	Postmortem tissues
R	77/F	-Shortness of breath -Dyspnea -Cough -Fatigue -Fever	-Ischemic cardiomyopathy -Hypertension -Type II diabetes mellitus -NASH^1^ -Dementia -NHC/B^2^	-BGGO^3^ -Kaletra^4^, chloroquine, Ceftriaxone	Increased creatinine levels, proteinemia, albuminemia	18 days	Trachea Lung, Kidney, Liver
J	70/M	-Dyspnea -Fever	-Ischemic cardiomyopathy -Hypertension -Coronary artery disease -Dementia -NHC/B	-BGGO -Kaletra, interferon alfa-2b, Azithromycin	Increased creatinine levels	15 days	Lung, Kidney
D	85/M	-Dyspnea -Fever	-Coronary artery disease -Hypertension -Dementia -NHC/B	-BGGO --Kaletra, interferon alfa-2b, Azithromycin	Increased creatinine levels	15 days	Lung, Kidney
B	68/F	-Shortness of breath -Dyspnea -Cough	-Coronary artery disease -Asthma -Obesity -NASH - Hypertension -NHC/B	-BGGO -Kaletra, interferon alfa-2b, Azithromycin	Increased creatinine levels, proteinemia, albuminemia, Increased liver damage markers	13 days	Lung, Kidney, Liver
T	80/F	-Dyspnea -Cough -Fatigue	-Coronary artery disease -Hypertension	-BGGO -Kaletra, interferon alfa-2b, Azithromycin		15 days	Lung
C (No COVID)	75/M		-Chronic obstructive pulmonary disease -Hypertension -Coronary artery disease		-	-	Lung

^1^Nonalcoholic steatohepatitis

^2^No history of HCV/HBV infection

^3^Bilateral ground glass opacity

^4^Lopinavir, ritonavir

### Procurement of specimens

With permission from the patient’s family, a limited autopsy to collect postmortem specimens [6] was performed in all cases by the autopsy service of the pathology department from the Hospital “Luis Díaz Soto” of Havana. This study received approval from the Ethics Committees of Hospital “Luis Díaz Soto” and the Center for Genetic Engineering and Biotechnology (CIGB). Postmortem tissue samples were obtained from visceral organs, including the lungs, liver, and kidneys within 3 hours after death. Autopsy was performed following recommendations and guidance on postmortem examinations of COVID-19 cases [38] and procedures established by the Cuban Health Ministry. Tissues were fixed with 4% paraformaldehyde in PBS for 1 hour and then routinely processed under standard biosafety conditions. To prepare frozen sections, tissues were washed with 20% sucrose in PBS overnight and then embedded in Tissue-Tek OCT compound (Sakura FineTek, Cat #4583, Tokyo, Japan). Ten-µm frozen sections were used.

### Masson’s trichrome and Picro Mallory staining

Staining of frozen lung and kidney sections with Masson’s trichrome and Picro Mallory stain was performed as described elsewhere [57, 122]. Staining was quantified using the open-source image processing package Fiji (National Institute of Health).

### Immunofluorescence staining and confocal microscopy

Immunofluorescent staining and confocal microscopy analysis of frozen lung, kidney, and liver sections was done as described previously with minor modifications [25, 26]. Tissue sections were washed with PBS, permeabilized with 0.5% Tween 20 (T20) in PBS (PBS+T20 0.5%), and saturated with 2% BSA in PBS+T20 0.1%, for 30 min. To detect the SARS-CoV-2 NC, sections were incubated with either mouse monoclonal IgG antibody (SINO Biologicals, catalog no. 40143-MM05) or SARS-CoV-2 NC peptide-specific (PKKDKKKKADETQALPQRQKK) [41] rabbit polyclonal antibodies or mouse monoclonal IgG antibody (CIGB Sancti Spíritus, Cuba; catalog no. CBSSNCov.2) for 2 hours at 27 °C (1:200 dilution in PBS+T20 0.1%). In addition, the following primary antibodies were used (1:100-200 dilutions in PBS+T20 0.1%): rabbit polyclonal anti-fibronectin (Dako Omnis, Agilent; catalog no. A0245), mouse monoclonal anti-microtubule-associated protein 2 (MAP2; Sigma; catalog no. M2320), rabbit polyclonal anti-ACE2 (a kind gift from the Center of Molecular Immunology, Havana, Cuba), rabbit polyclonal anti-DDX3X (a kind gift from A.H. Patel, MRC-University of Glasgow Centre for Virus Research, Glasgow, UK), rabbit polyclonal anti-NG2 (chondroitin sulphate proteoglycan 4 [CSPG4]; Abcam; catalog no. Ab81104), rabbit polyclonal anti-phospho S112 peroxisome proliferator activated-receptor γ (PPARγ; Abcam; catalog no. Ab60953), rabbit polyclonal anti-prohibitin (PHB; Abcam; catalog no. Ab 75766), rabbit polyclonal anti-PGC1 (Abcam; catalog no. Ab 72230), mouse monoclonal anti-keratin 10 (K10) (Thermo Scientific, catalog no. MA1-35857), mouse monoclonal anti-CD34-FITC conjugate (Dako, catalog no. F7081), mouse monoclonal IgG anti-CD68 (Dako Omnis, Agilent; catalog no. M0814), mouse monoclonal anti-CD163-FITC conjugate (Pharmigen, catalog no. 563697), mouse monoclonal anti-CD163-APC conjugate (Invitrogen, catalog no. 17-1639-42), mouse monoclonal anti-IL1β-FITC conjugate (Invitrogen, catalog no. 11-7018-42), mouse monoclonal anti-PD1-APC conjugate (Invitrogen, catalog no. 17-2799-42), mouse monoclonal anti-CD47-FITC conjugate (Biolegend, catalog no. 323106), mouse monoclonal anti-PDL1-APC conjugate (Invitrogen, catalog no. 17-5983-42), mouse monoclonal anti-IL6-PE conjugate (Invitrogen, catalog no. 12-7069-82), and mouse monoclonal anti-vimentin (VMT) (Sigma Aldrich, catalog no. V6389). After washing with PBS+T20 0.1%, slides were incubated for 1 hour at 27 °C with one of the following secondary antibodies: fluorescein-conjugated goat anti-rabbit IgG (KPL; catalog no. 172-1506) or goat anti-mouse IgG (KPL; catalog no. 02-18-18), Alexa Fluor 647–conjugated either anti-mouse IgG (Cell Signalling; catalog no. 4410S) or anti-rabbit IgG (Cell Signalling; catalog no. 4414S), Alexa Fluor 594–conjugated anti-rabbit IgG (Cell Signalling; catalog no. 8889) (1:250-500 dilution in PBS+T20 0.1%, depending on the combination of primary and secondary antibodies used). When required, lipid droplets (LDs) were stained using Oil Red O for 30 min and washed with water. Some negative controls included sections that were incubated directly with Alexa 647– or fluorescein-conjugated goat anti-mouse/rabbit IgGs. Subsequently, samples were washed with PBS+T20 0.1%, nuclei were counterstained with 4′,6-diamidino,2-phenylindole (DAPI) (1 mg/mL) (KPL; catalog no. 1-03-01, Gaithersburg, USA), and the preparation was coverslipped in Vectashield mounting medium (Vector Laboratories, catalog no. H-1000, Burlingame, CA., USA). Samples were analyzed using an Olympus FV1000 IX81 laser scanning fluorescence microscope (Olympus Corporation, Japan) and the imaging software FlowView Viewer v3.1. Images were also taken with differential interference contrast (DIC) microscopy. Channels were recorded sequentially, and images were acquired as z-stack series. Images with a field of view of 512×512 pixels, 621,000.0 µm/pixel, were acquired with a sampling speed of 20000.0 µs/pixel. All images were taken with a bit depth of 12 bits. Colocalization between the different channels (described in Supplementary methods) was analyzed from image stacks using the open-source image processing package Fiji with Just Another Colocalization Plugin (JACoP) [10].

## Results

### Analysis of postmortem lung samples

Both clinical and pathology evidence indicated the development of ARDS in all patients [27]. The pulmonary tissue showed evidence of a distinctive diffuse acute alveolar damage (DAD) pattern with predominant advanced phases (fibro-proliferative and fibrotic phases) (Supplementary Fig. S1, representative results illustrated for patient R). Lung remodeling with typical interstitial fibrosis showing features of fibrosis by accretion was the most important pathological mechanism identified in all patients. Alveolar damage with destruction of the alveolar wall lining with desquamation of alveolar type I pneumocytes (AT1) and type 2-like pneumocytes (AT2) proliferating along the surface of fibrous alveolar septa were frequently observed (Supplementary Figs. S1 and S2A). On the other hand, SARS-CoV-2 isolated from nasal swaps from patient R was grown in cell culture (Vero E6 cells), where numerous virus-like particles (VLPs) (ranging from 80 nm to 125 nm in diameter) could be seen at low magnification (Supplementary Fig. S3). In addition, aged hamsters could be infected by cell-culture-adapted SARS-CoV-2 (Supplementary Fig. S4) pointing to the infectious nature of these viral isolates. Interestingly, infected hamsters developed features of pulmonary fibrosis (Supplementary Fig. S2B).

**Fig. 1:**
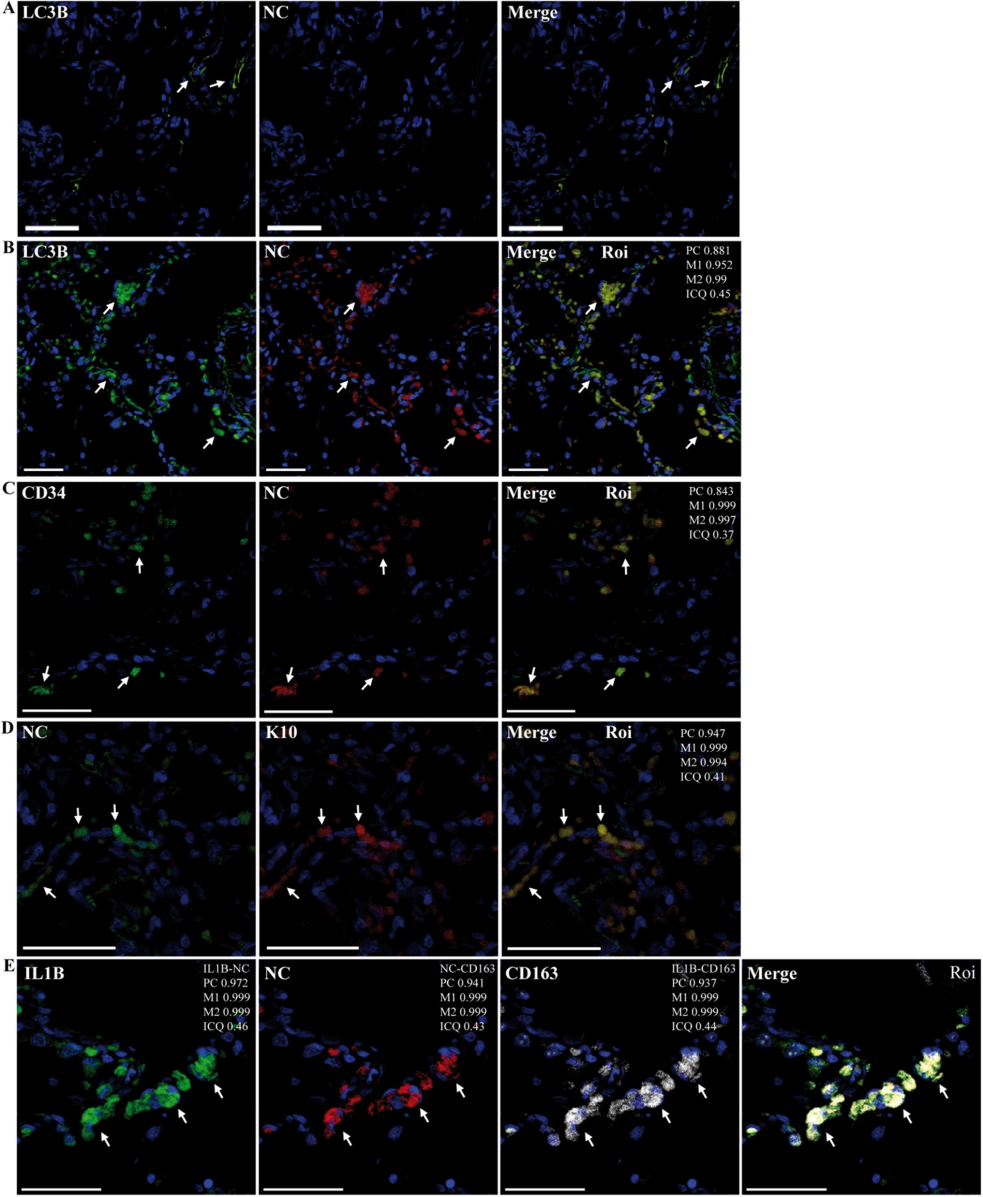
Representative confocal microscopy images of lung sections from a person who died from a cause unrelated to COVID-19 and from patient R, incubated with various combinations of rabbit and mouse (CIGB, Sancti Spíritus) antibodies against NC and host proteins, followed by Alexa 647 (A647)- and fluorescein/(FITC)-conjugated anti-mouse/rabbit IgGs in different combinations, or other host-protein-specific (IL1β, CD163) primary mouse monoclonal antibodies conjugated to either FITC or APC. DAPI was used to stain the nucleus (blue channel). Colocalization was quantified using calculated intensity correlation quotients (ICQ) and Pearson’s (PC) and Manders’ (M1, M2) coefficients (see Supplementary Fig. S6). Bars: 50 µm. (**A**) Lung section of a person who died from a cause unrelated to COVID-19, showing no staining for NC (A647). As a reference, a mouse monoclonal antibody against LC3B was used (FITC; arrows) (20X magnification). (**B**–**E**) Illustrative regions of interest (ROIs) of lung sections from patient R showing colocalization between NC (A647) and LC3B (FITC) (**B**) (20X magnification) localization of NC (FITC or A647) with CD34^+^ (FITC) (**C**) or K10^+^ (**D**) cells and, concomitant with CD163^+^ (APC) and IL1β (FITC). Arrows indicate positive co-staining (40X magnification)

Next, SARS-CoV-2 was detected in samples using confocal microscopy and antibodies specific for the NC protein of SARS-CoV-2 as described previously [25, 41, 61]. First, primary anti-NC antibodies were detected in lung samples from SARS-CoV-2-infected hamsters. As shown in Supplementary Fig. S4A-C, NC was detected (by all of the primary anti-NC antibodies used) in the lungs of SARS-CoV-2-infected hamsters, but not in those of mock-infected animals. The presence of SARS-CoV-2 was then analyzed in postmortem lung samples. While SARS-CoV-2 NC could not be detected in lung samples from patient T or from a non-COVID-19 patient who died after suffering from chronic obstructive pulmonary disease (COPD) (Fig. 1A, Supplementary Fig. 4D, 5A), it was identified in lung sections from patients R, J, D, and V (Fig. 1, Supplementary Figs. S4E and F and S5). These results indicated the presence of SARS-CoV-2 in the lungs of patients R, J, D, and B, but not of patient T, at advanced stages of DAD.

**Fig. 2:**
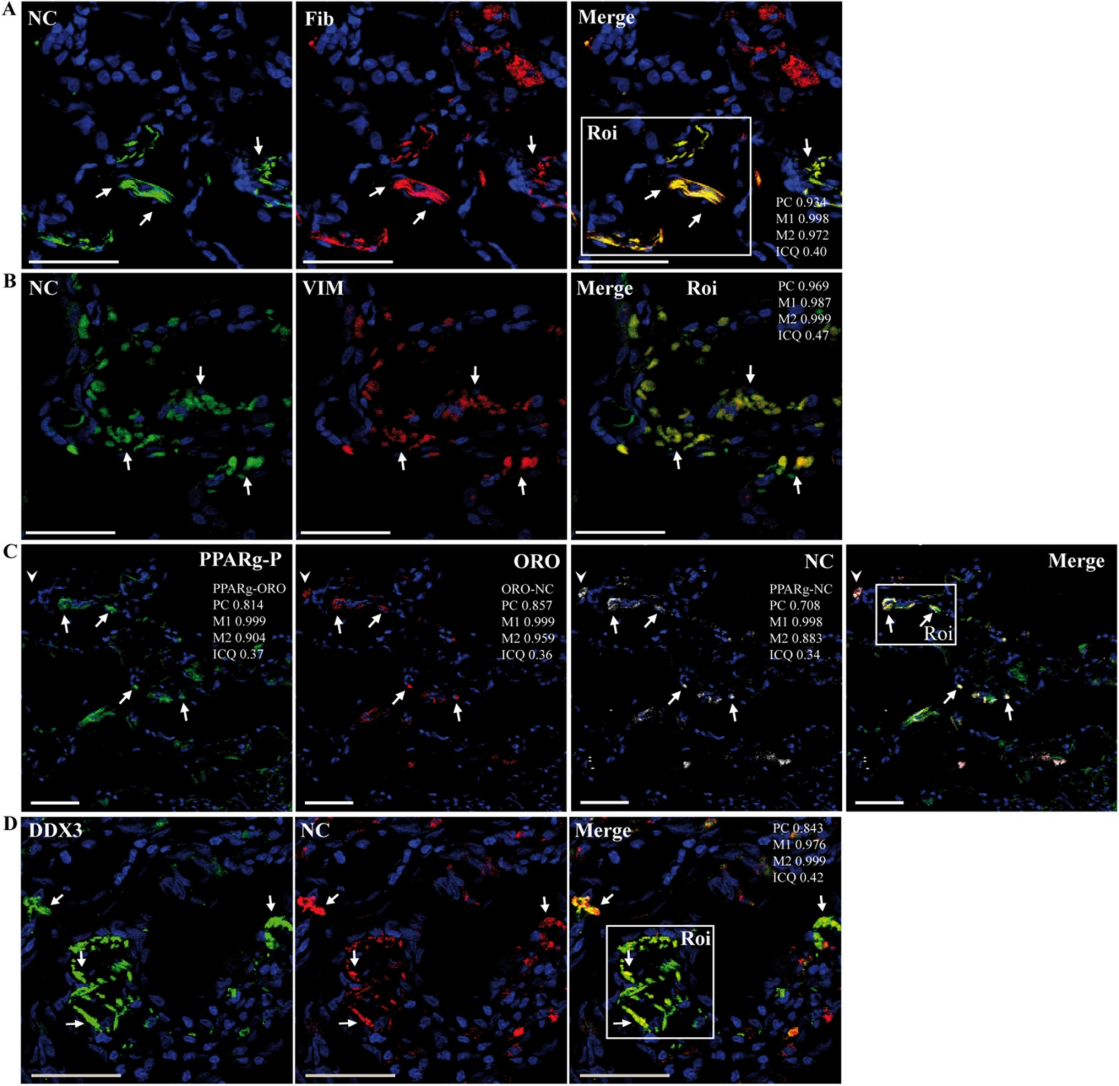
Representative confocal microscopy images of lung sections from patient R incubated with various combinations of rabbit or mouse (CIGB, Sancti Spíritus) antibodies against NC and anti-fibronectin, anti-VMT, anti-DDX3X, or anti-phospho S112 PPARγ (PPARγ-P) antibodies, followed by fluorescein- (FITC) or Alexa 594/647 (A594/647)-conjugated anti-rabbit/mouse IgGs) or stained with Oil Red O (ORO, TxRed channel). DAPI was used to stain the nucleus (blue channel). Colocalization was quantified using calculated intensity correlation quotients (ICQ) and Pearson’s (PC) and Manders’ (M1, M2) coefficients (see Supplementary Fig. S7). Bars: 50 µm. (**A**–**D**) Illustrative ROIs of lung sections showing NC (FITC) detected in fibronectin^+^ cells (A647) (**A**) and VMT^+^ (VIM) cells (A647) (**B**) (40X magnification); NC (A647) detected in cells showing concomitant LDs (ORO) and PPARγ-P (FITC) (**C**) (20X magnification) colocalization of NC (FITC) and DDX3X^+^(A647) (**D**) (40X magnification). Arrows indicate positive co-staining

Interestingly, a striking colocalization of NC and LC3B was observed, indicating the possible involvement of autophagic functions during SARS-CoV-2 infection (Fig. 1B). NC localized to alveolar epithelial cells, endothelial-like cells (ECLs), and macrophage-like cells (MLCs) (Figs. 1, 2, and 4; Supplementary Figs S5-S9). Markers of endothelium and endothelial progenitor cells (CD34) [88, 114] (Fig. 1C), bronchial and alveolar epithelial cells (K10) [87] (Fig. 1D), and monocyte/macrophages (CD163) [14] (Fig. 1E; Supplementary Figs. S5-S9)/(CD68, [25]) showed that NC was present in these cell types. NC was detected in both alveolar and interstitial CD163^+^ MLCs [14] (Supplementary Figs. S5C and S8B). In agreement with our previous study showing activation of NLRP3 in lung macrophages containing SARS-CoV-2 [25], NC was detected in CD163^+^ MLCs showing expression of IL1β (Fig. 1E; Supplementary Fig. S6D).

In addition, NC staining was found on ACE2^+^ cells (Supplementary Figs. S5B andS8A), and interestingly, NC also displayed a staining pattern indicating its presence in the interface of the capillary endothelium and alveolar epithelial cells, along the inside of the alveolar septum, and surrounding the blood vessels representing the connective tissue, including fibroblast-like cells (FLCs) (Supplementary Fig. S5D). In addition, analysis of tracheal sections from patient R showed the presence of SARS-CoV-2 NC in the connective tissue and FLCs (Supplementary Fig. S5D). To investigate possible interactions of SARS-CoV-2 with extracellular matrix (ECM) components and FLCs, we performed double immunofluorescence staining of NC with key molecular targets involved in the wound healing response and lung pathogenesis. First, co-staining of NC and fibronectin was studied. As shown in Fig. 2A, NC was co-detected with fibronectin in the alveolar septa, suggesting its presence in fibronectin-expressing cells, including FLCs, and also in the ECM (Fig. 2A; Supplementary Fig. S7A). Then, VMT (major type III intermediate filament cytoskeletal protein of mesenchymal cell origin, including FLCs), which is also expressed in alveolar epithelial cells undergoing epithelial-to-mesenchymal transition (EMT) during injury repair [47], was studied. Notably, NC was strongly co-detected with VMT (Fig. 2B; Supplementary Fig. S7B), suggesting the presence of SARS-CoV-2 in the connective tissue and associated cells. Subsequently, the presence of SARS-CoV-2 in lipofibroblast-like cells (LPFs), a critical cell type for lung homeostasis and injury repair, was investigated. LPFs are the main LD-producing cells in the alveolar interstitium [71, 72], and therefore, the presence of LDs and expression of PPARγ are key features of LPFs [71, 72]. Notably, NC was detected in interstitial cells showing the simultaneous presence of LDs and activated (phosphoS112) PPARγ, suggesting the presence of SARS-CoV-2 in LPFs displaying PPARγ signaling (Fig. 2C; Supplementary Fig. S7D). On the other hand, DDX3X, a host protein involved in the life cycle of various viruses that has been shown to be recruited to LDs during HCV infection [4], was co-detected with NC (Fig. 2D; Supplementary Fig. S7C), and DDX3X also colocalized with LDs (not shown).

**Fig. 3:**
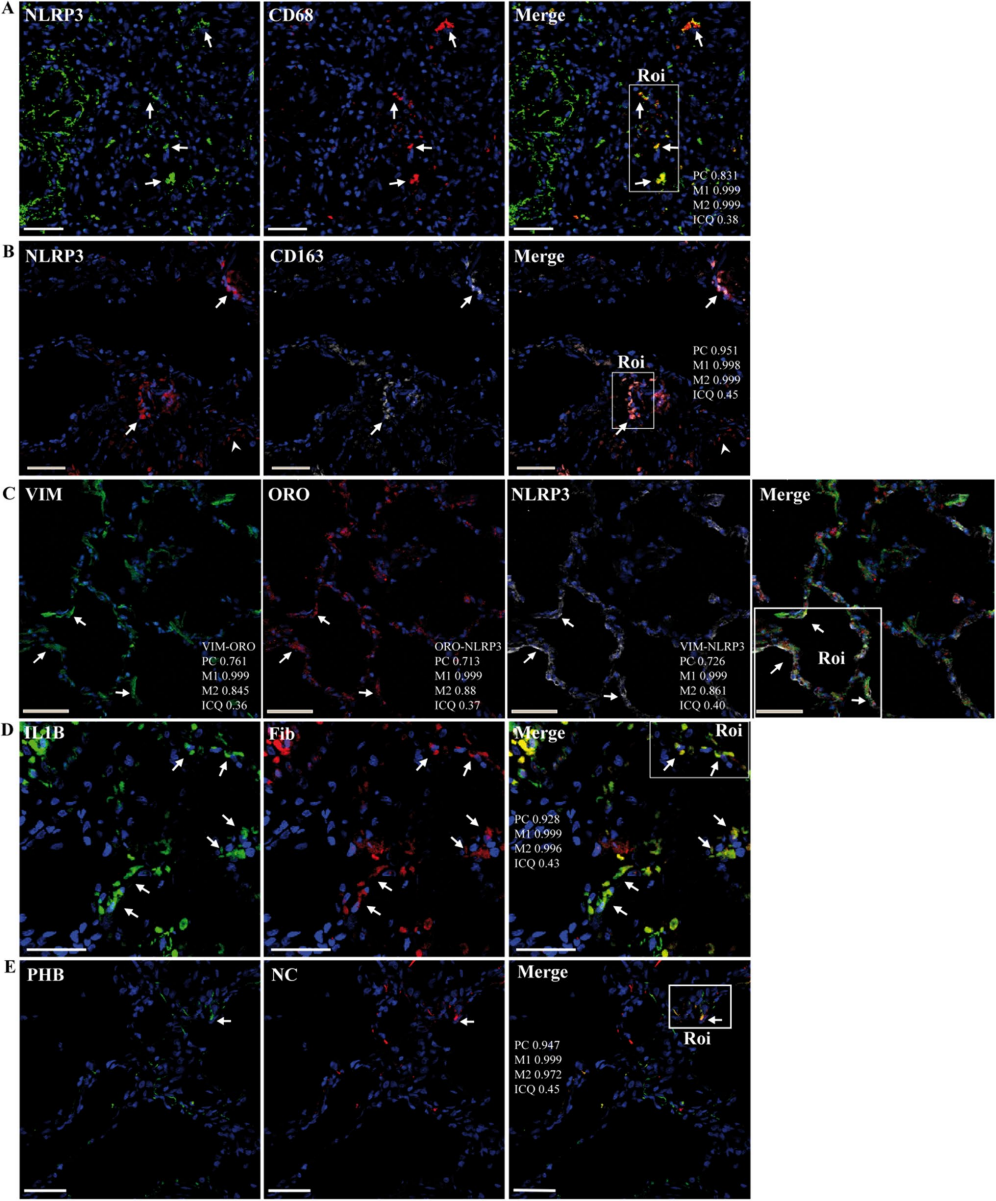
Representative confocal microscopy images of lung sections from patient R incubated with various combinations of rabbit and mouse antibodies against NC, fibronectin, VMT, CD68, NLRP3, and PHB, followed by fluorescein- (FITC) or Alexa 594/647 (A594/647)-conjugated anti-rabbit/mouse IgG or host-protein-specific (IL1β, CD163) primary mouse monoclonal antibodies conjugated to either FITC or APC, respectively; or stained with Oil Red O (ORO, TxRed channel). DAPI was used to stain the nucleus (blue channel). Colocalization was quantified using calculated intensity correlation quotients (ICQ) and Pearson’s (PC) and Manders’ (M1, M2) coefficients (see Supplementary Fig. S8C-G). Bars: 50 µm. (**A**–**D**) Illustrative ROIs of lung sections showing NLRP3 (FITC) localized to either CD68^+^ (arrows) or CD68^-^ cells (arrowheads) (A647) (**A**) (20X magnification); NLRP3 (A594) localized to either CD163^+^ (arrows) or CD163^-^ cells (arrowheads) (APC) (**B**); concomitant localization of VMT (VIM)(FITC) with ORO (TxRed) and NLRP3 (A647) (**C**); IL1B localized to Fib-expressing cells (**D**) (40X magnification). Arrows indicate positive co-staining.

Interestingly, NLRP3 staining was observed not only in CD68^+^ and CD163^+^ cells but also in CD68^-^ and CD163^-^ interstitial cells (Fig. 3A and B, Supplementary Fig. S8C and D). Given that FLCs are involved in the inflammasome-mediated response [77] and that VMT has been shown to play a key role in biogenesis of LDs and activation of NLRP3 [24, 40], we investigated the colocalization of these proteins. Interestingly, NLRP3 partially colocalized to alveolar interstitial cells showing concomitant expression of vimentin and ORO staining (Fig. 3C, Supplementary Fig. S8F), suggesting that LD-containing cells possibly representing LPFs may be involved in the inflammasome response. IL1β staining was also observed in fibronectin-expressing cells (Fig. 3D, Supplementary Fig. S8E). We noted frequent mitochondrial damage in the samples analyzed by electron microscopy (Supplementary Fig. S2, S11, and S14). As mitochondrial damage is related to NLRP3 activation through mitochondrial reactive oxygen species (mtROS) and oxidized mitochondrial DNA (mtDNA), which are increased in airway macrophages in cases of pulmonary fibrosis [99], we searched for colocalization of NC with proteins that are commonly recruited or present in mitochondria, including prohibitin (PHB) and PGC1α [5, 89]. As illustrated in Fig. 3E, NC colocalized with PHB in some cells (Fig. 3, Supplementary Fig. S8F), raising the possibility of direct virus-mediated mitochondrial impairment.

**Fig. 4:**
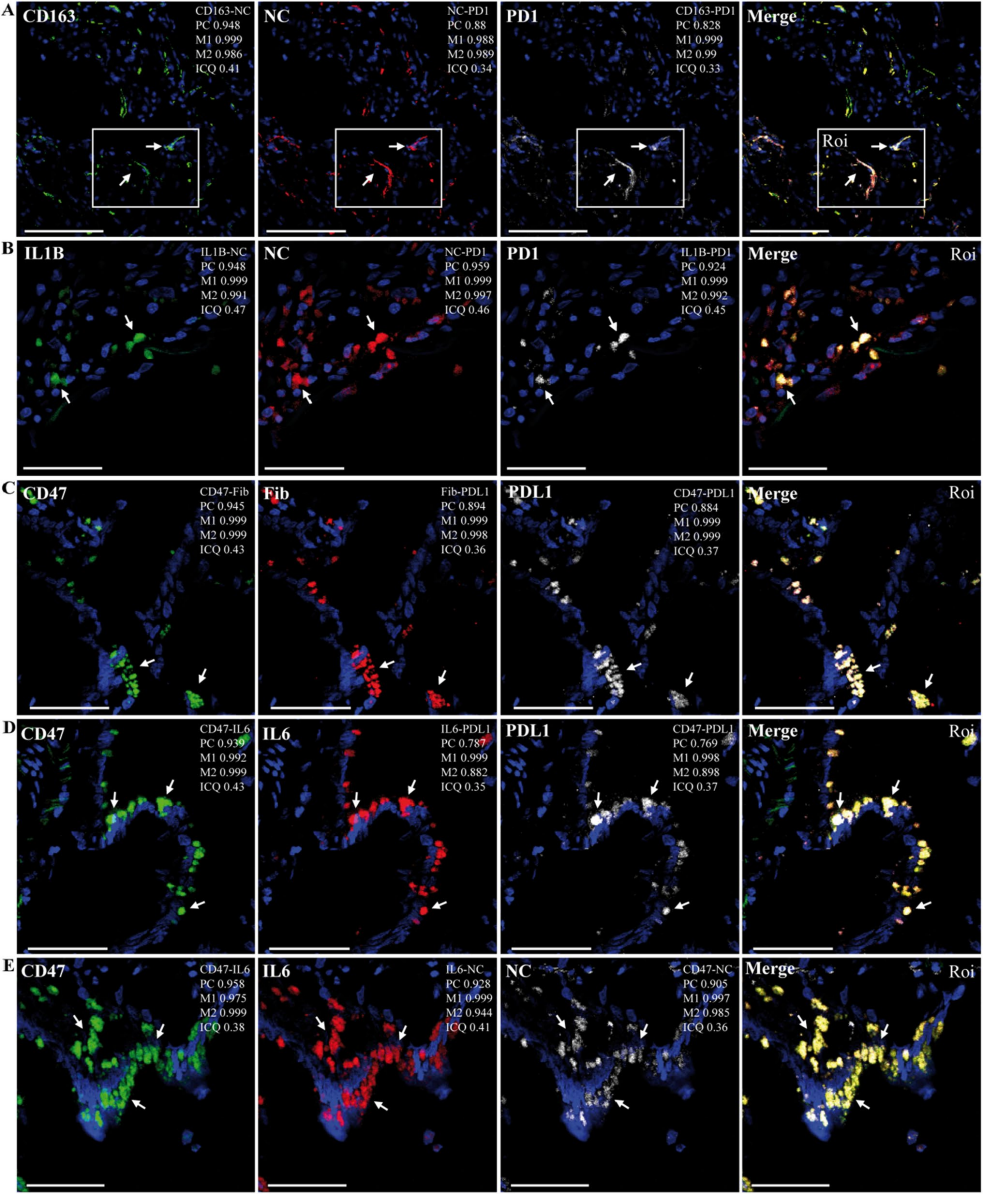
Representative confocal microscopy images of lung sections from patient R incubated with various combinations of rabbit polyclonal antibodies against NC or fibronectin, followed by Alexa 594/647 (A594/647)-conjugated anti-rabbit IgG and host protein-specific (IL1β, CD163, PD1, CD47, IL6, PDL1) primary mouse monoclonal antibodies conjugated to FITC, PE, or APC. DAPI was used to stain the nucleus (blue channel). Colocalization was quantified using calculated intensity correlation quotients (ICQ) and Pearson’s (PC) and Manders’ (M1, M2) coefficients (see Supplementary Fig. S9). Bars: 50 µm. (**A**–**E**) Illustrative ROIs of lung sections showing NC (A594) detected in PD1^+^ cells (APC) concomitantly with either CD163 (**A**) or IL1β (**B**) (FITC) (arrows) (20X and 40X magnification, respectively); Fib (A594) (**C**) or IL6 (PE) (**D**) detected concomitantly with CD47 (FITC) and PDL1 (APC) (arrows); NC (A647) detected in CD47^+^ cells (FITC) concomitantly with IL6 (PE) (**E**) (arrows) (40X magnification)

Previous studies have shown that populations of lung-fibrotic fibroblasts (expressing JUN and IL6 with upregulation of the immune-checkpoint proteins CD47 and PDL1) and immunosuppressive PD1^+^ macrophages (expressing IL1β) are involved in impaired alveolar regeneration and a weakened adaptive T cell immune response during pulmonary fibrosis in humans and mice, as well as during SARS-CoV-2 infection [18, 19, 21, 59]. Given our evidence for impaired epithelial regeneration, induction of IL1β, and the presence of SARS-CoV-2 NC in FLCs and MLCs, we next studied the occurrence of NC in CD47^+^ PDL1^+^ IL6^+^ and CD163^+^ PD1^+^ cells. As shown in Fig. 4A, NC was detected concomitantly with CD163 and PD1, indicating its presence in regulatory CD163^+^ PD1^+^ macrophages (Supplementary Fig. S9A). Interestingly, NC was also co-detected with PD1 and IL1β, suggesting that regulatory PD1^+^ macrophages containing NC were able to produce IL1β (Fig. 4B, Supplementary Fig. S9B). In addition, CD47 and PDL1 were identified simultaneously with fibronectin (Fig. 4C, Supplementary Fig. S9C) as well as with IL6 (Fig. 4D, Supplementary Fig. S9D), indicating the presence of CD47^+^ PDL1^+^ FLCs able to produce IL6. Notably, NC was also found together with CD47 and IL6 (Fig. 4E, Supplementary Fig. S9E), suggesting the presence of SARS-CoV-2 in lung-fibrotic FLCs.

### Analysis of postmortem kidney samples

**Fig. 5:**
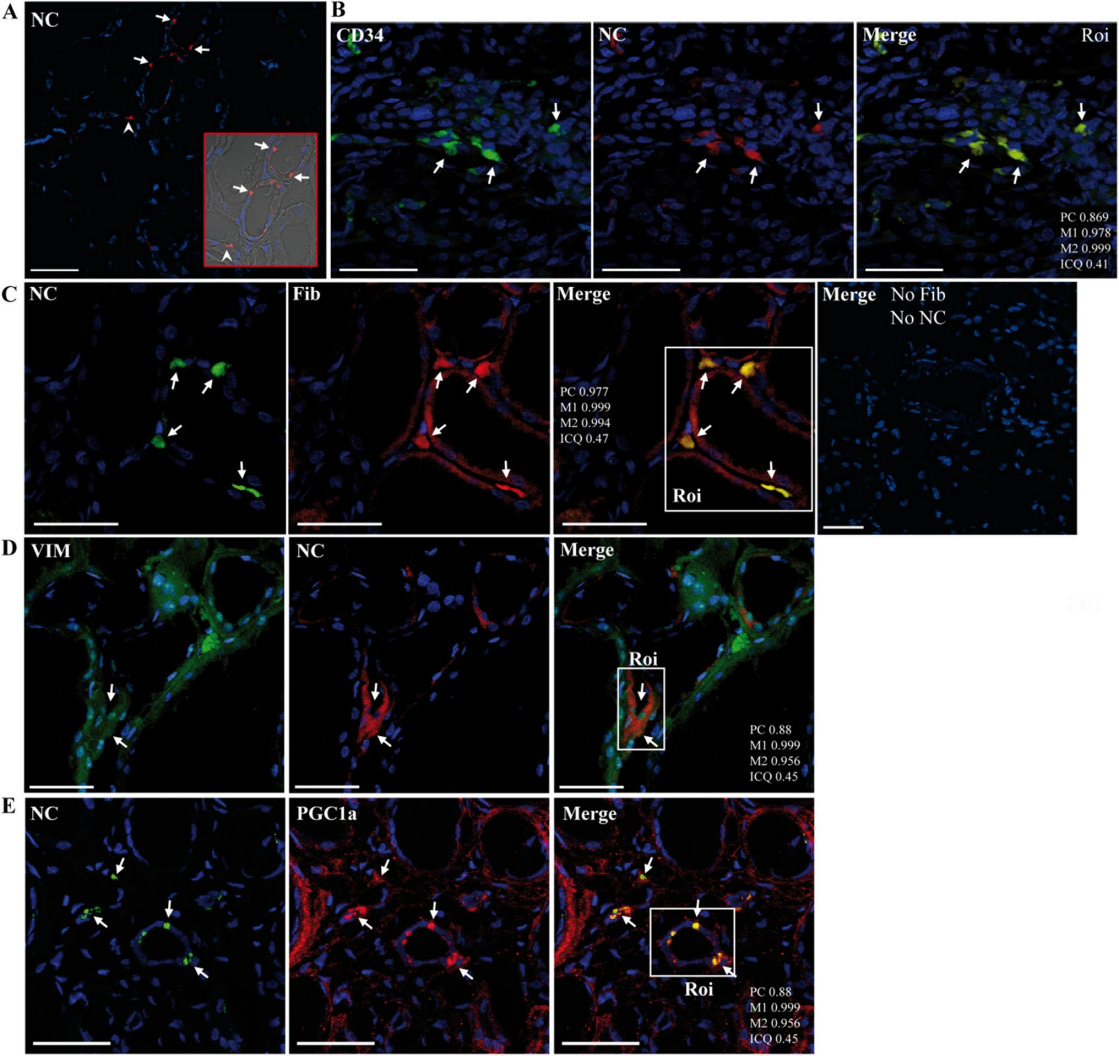
Representative confocal microscopy images of kidney sections from patient R incubated with various combinations of rabbit or mouse (CIGB, Sancti Spíritus) antibodies against NC and host proteins, followed by Alexa 647 (A647)- and fluorescein/FITC-conjugated anti-mouse/rabbit IgGs, either alone or in different combinations. DAPI was used to stain the nucleus (blue channel). Bars: 50 µm. (**A**) Renal cortex section showing detection of NC (A647) in tubule epithelial cells (arrows) and peritubular interstitial cells (arrowhead) (20X magnification). (**B**) Renal medullary section showing that NC (A647) localized to the endothelium of CD34^+^ vessels and interstitial cells (arrowheads) (40X magnification). **C**–**E**) Renal cortex sections showing localization of NC (FITC) to Fib^+^ peritubular interstitial cells (A647) (arrows) (40X magnification). Note the negative control of a section incubated only with secondary fluorescent-probe-conjugated antibodies without primary antibodies (Merge, No Fib, No NC) (20X magnification). (**C**) NC (A647 or FITC) localized to VMT^+^ (VIM) cells (FITC) (arrows) (**D**) and colocalized with PGC1α (arrows) (**E**) (40X magnification)

Pathological features observed in kidney samples included acute tubule injury and interstitial fibrosis (Supplementary Fig. S10A and S11) [15]. Some glomeruli were shrunken with widened Bowman space. There was also some occlusion of the microvascular lumen in peritubular and glomerular capillary loops. Damaged mitochondria were commonly observed. Some VLPs ranging from 60 nm to 84 nm in diameter were found in a proximal tubule cell (Supplementary Fig. S11). However, as these are low-magnification electron microscopy images, these VLPs lacked sufficient ultrastructural detail to be identified as SARS-CoV-2-related particles. Immunofluorescence analysis of SARS-CoV-2 showed patchy granular cytoplasmic staining of NC in tubular epithelial cells (Fig. 5A). Importantly, NC staining in the juxtaglomerular apparatus was also observed (Supplementary Fig. S10B1, B2 and C). In addition, NC could be detected in podocytes, mesangial cells, and endothelial cells in some glomeruli (Supplementary Fig. S10B3). Moreover, NC was found in the medullar region, in CD34^+^ endothelium of vessels, and in interstitial cells (Fig. 5B, Supplementary Fig. S12A). Interestingly, NC localized to peritubular fibronectin^+^ interstitial cells and also to some VMT^+^ cells (Fig. 5A, C, and D; Supplementary Fig. S12B, and C). In addition, NC staining colocalized with PGC1α (Fig. 5E, Supplementary Fig. S12D) and PHB (not shown), providing further support for the presence of NC in or near mitochondria.

**Fig. 6:**
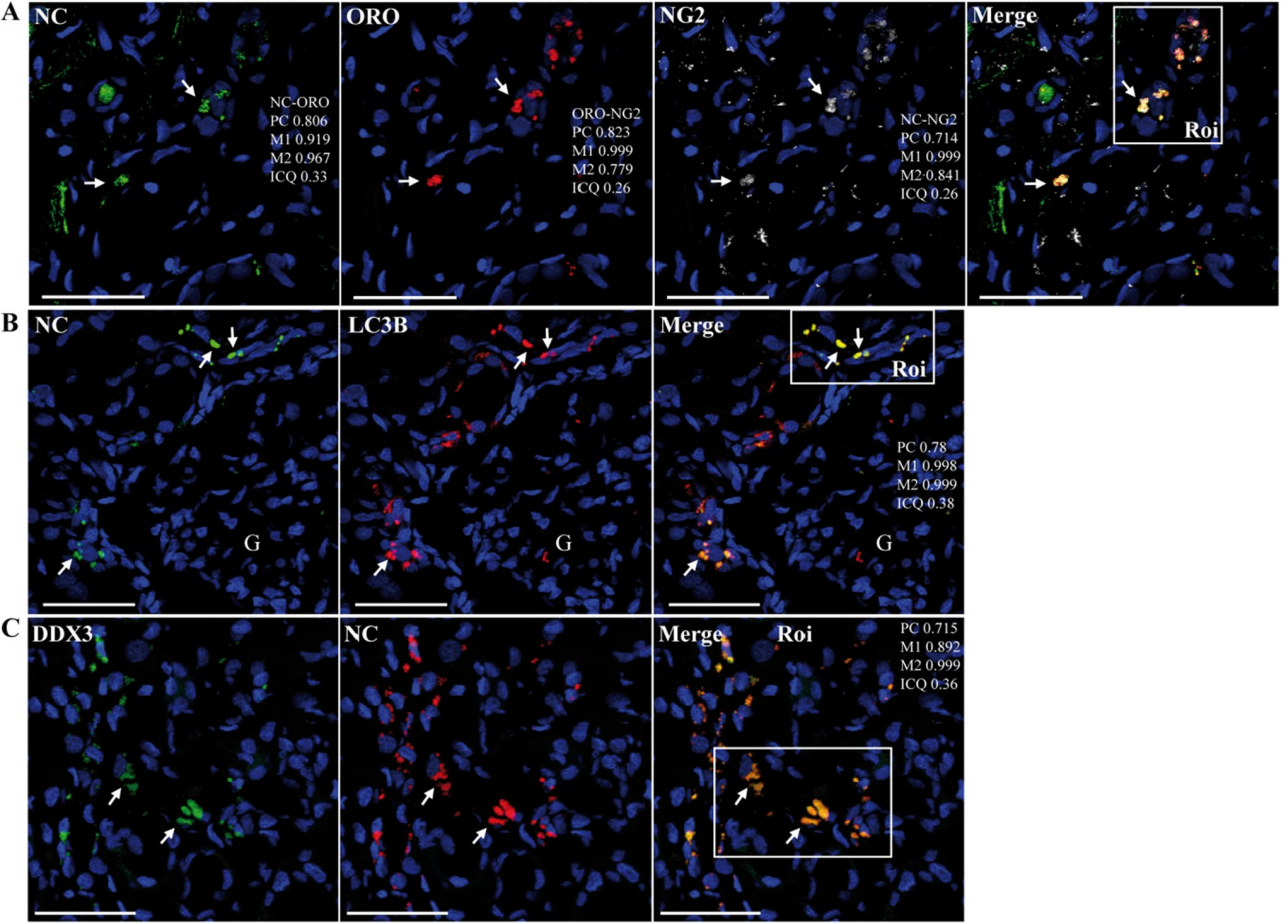
Representative confocal microscopy images of kidney sections from patient R incubated with various combinations of rabbit and mouse (CIGB, Sancti Spíritus) antibodies against NC and host proteins, followed by Alexa 647 (A647)- and fluorescein/FITC-conjugated anti-mouse/rabbit IgG, either alone or in different combinations, or stained with Oil Red O (ORO, Tx Red channel). DAPI was used to stain the nucleus (blue channel). Bars: 50 µm. (**A**) Renal medullary section showing that NC (A647) localized to NG2^+^ cells displaying LDs (ORO) (arrows). (**B**) Renal cortex section showing colocalization between NC (FITC) and LC3B (A647). G, glomerulus. (**C**) Colocalization between DDX3X (FITC) and NC (A647) in a renal medullary section (40X magnification)

A key feature of interstitial cells is the accumulation of LDs (Supplementary Fig. S11C) as well as the expression of NG2, especially in the medullar region [52]. Notably, NC was found in NG2^+^ cells displaying strong LD staining, further suggesting the presence of SARS-CoV-2 in renal interstitial cells (Fig. 6A, Supplementary Fig. S13A). NC localized to both renal tubular and interstitial ACE2^+^ cells (Supplementary Figs. S10D and S13B). Moreover, similar to what was observed in lung samples, NC colocalized with LC3B and DDX3X (Fig. 6B and C and Supplementary Fig. S13C and S13D).

### Analysis of postmortem liver samples

**Fig. 7:**
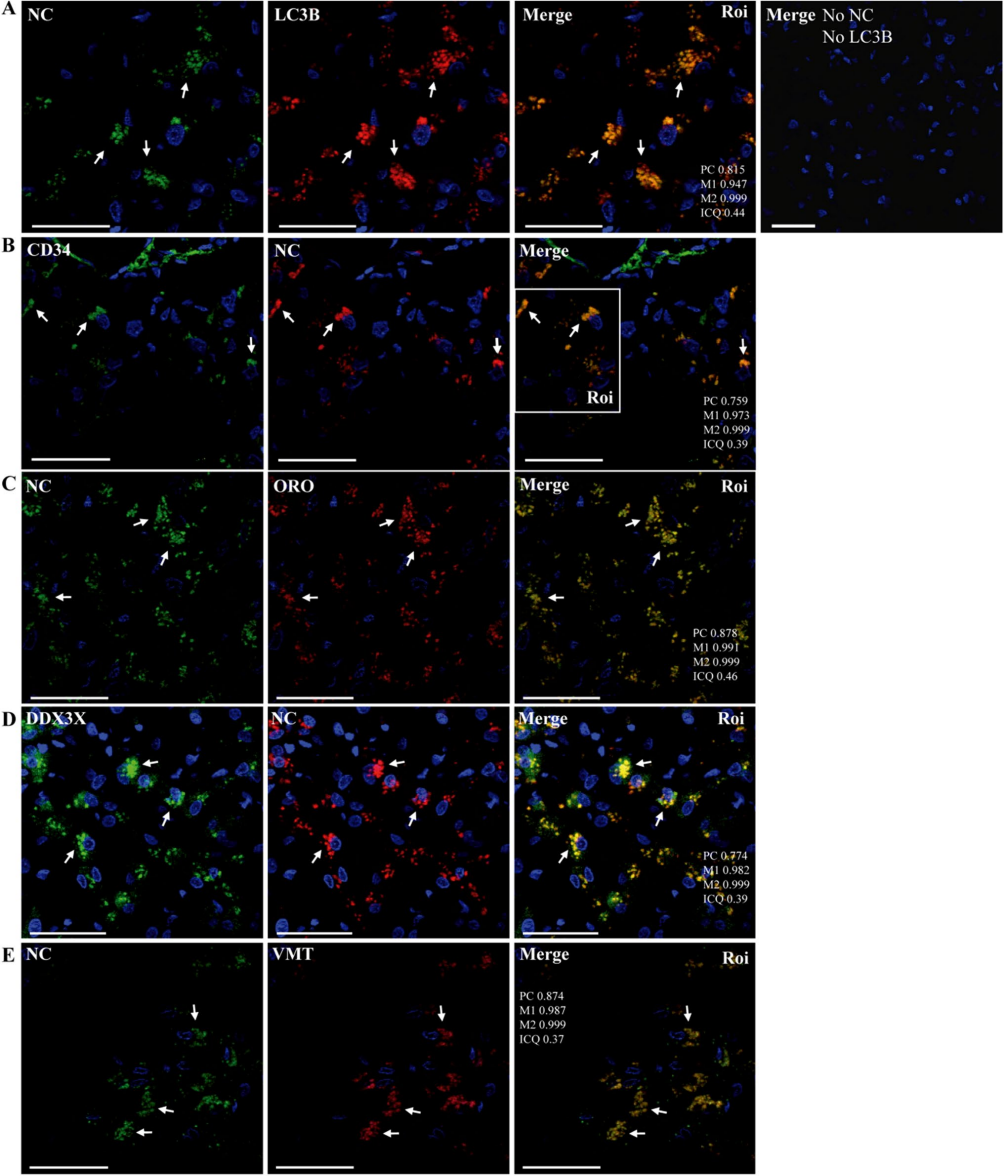
Representative confocal microscopy images of liver sections from patient R incubated with various combinations of rabbit and mouse (CIGB, Sancti Spíritus) antibodies against NC and host proteins, followed by Alexa 647 (A647)- and fluorescein/FITC-conjugated anti-mouse/rabbit IgG, either alone or in different combinations. DAPI was used to stain the nucleus (blue channel). Bars: 50 µm. (**A**–**E**) Illustrative ROIs of liver sections from patient R showing and colocalization between NC (FITC) and LC3B (A647) (**A**), LDs (ORO, TxRed) (**C**), VMT (A647) (arrows) (**E**); localization of NC (A647) in CD34^+^ cells (FITC) (arrows) (**B**); colocalization between NC (A647) and DDX3X (FITC) (arrows) (**D**) (40X magnification)

**Fig. 8:**
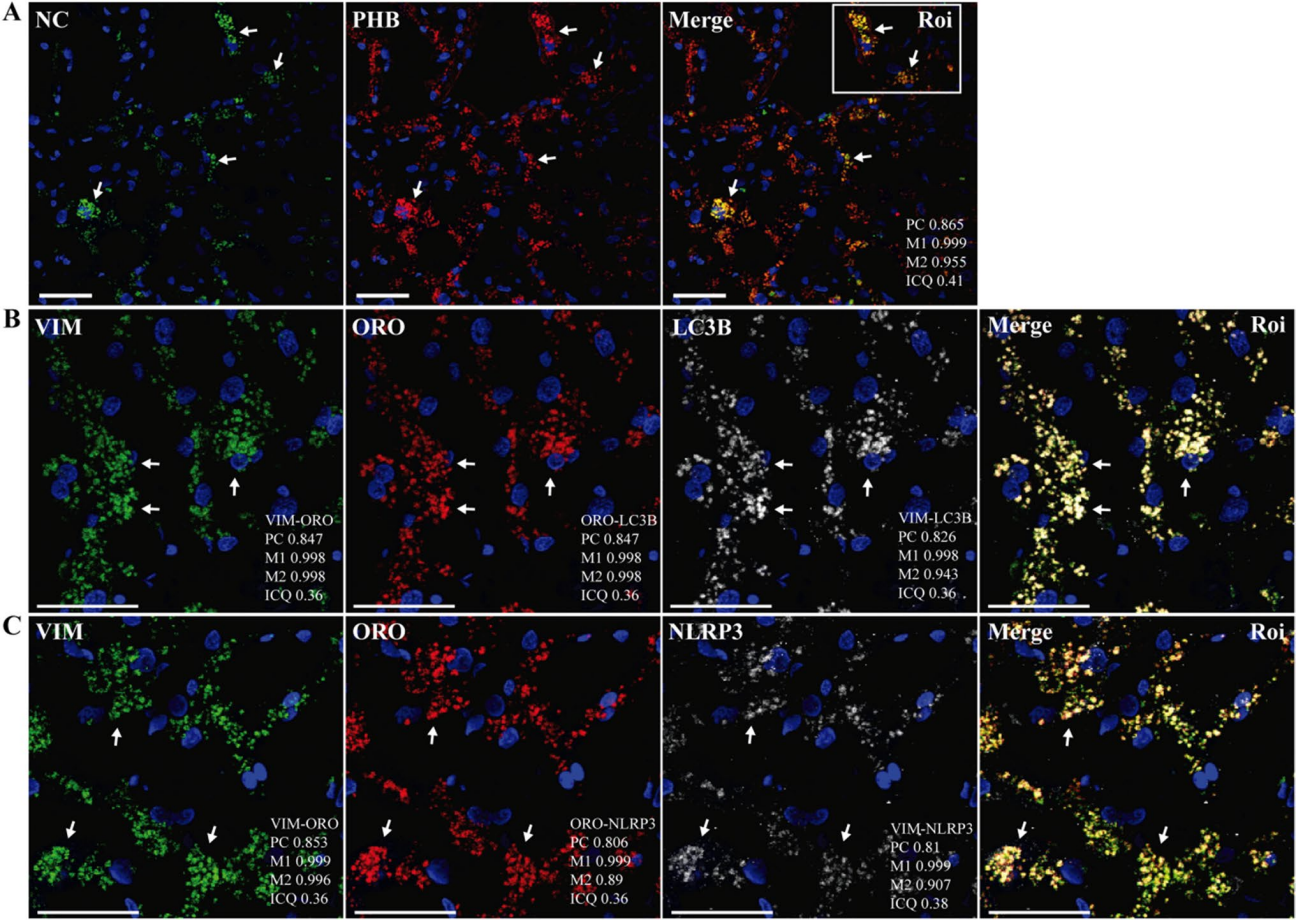
Representative images from confocal microscopy analysis of liver sections of patient R incubated with various combinations of rabbit and mouse antibodies against NC, PHB, VMT (VIM), LC3B, or NLRP3, followed by fluorescein- (FITC) and Alexa 647 (A647)-conjugated anti-rabbit/mouse IgG or stained with Oil Red O (ORO, Tx Red channel). DAPI was used to stain the nucleus (blue channel). Colocalization was quantified using calculated intensity correlation quotients (ICQ) and Pearson’s (PC) and Manders’ (M1, M2) coefficients (see Supplementary Fig. S13). Bars: 50 µm. (**A**–**C**) Illustrative ROIs of lung sections from patient R showing NC colocalized with PHB (arrows) (**A**), LC3B (A647) detected in VMT^+^ (FITC) cells showing LDs (ORO) (arrows) (**B**); and NLRP3 (A647) detected in VMT^+^ (FITC) cells showing LDs (ORO) (arrows) (**C**) (40X magnification)

Steatosis was the major pathological finding observed in liver samples [15]. In addition, clusters of small LDs in lipolysosome-like structures similar to those reported in hepatocytes [82] and in non-alcoholic fatty liver disease (NAFLD) patients [16], as well as damaged mitochondria, were frequently observed (Supplementary Fig. S14). Immunostaining of NC was detected in portal tracks including the connective tissue (Supplementary Fig. S15) and hepatocytes (Fig. 7, Supplementary Fig. S15). NC was also found in liver endothelial sinusoidal cells (LESCs) and adjacent hepatocytes, some of which showed ACE2 staining (Supplementary Figs. S15 and S16F). Interestingly, NC could be detected despite scarce ACE2 staining in liver samples from patient B (Supplementary Fig. S15C). Moreover, NC was detected in CD34^+^ cells, indicating the presence of SARS-CoV-2 in LSECs (Fig. 7B, Supplementary Fig. S16B). Furthermore, NC showed strong co-staining with LC3B, LDs, DDX3X, and VMT (Fig. 7; Supplementary Fig. S16). DDX3X (not shown) and LC3B (Fig. 8) also colocalized with LDs. This granulated staining pattern may indicate cellular redistribution of viral and host proteins to LDs and/or possibly viral replication-morphogenesis sites. Moreover, NC colocalized with PGC1α (Fig. 8A; Supplementary Fig. S16G) and PHB (not shown). These staining patterns prompted us to investigate the relationship of VMT with LDs, autophagy, and inflammasome markers in liver samples [9, 24, 40]. As shown in Fig. 8B and C (Supplementary Fig. S17), VMT localized together with LDs, LC3B, and NLRP3, suggesting its involvement in lipid metabolism, autophagy, and inflammasome functions.

## Discussion

### Findings in postmortem lungs

DAD is the pathological hallmark of ARDS [7]. SARS-CoV-2-mediated direct lung injury has been shown previously to be particularly relevant at early stages of infection, while later stages of DAD development have mostly been associated with host cellular responses [11, 79]. Advanced DAD with fibro-proliferation and fibrosis was seen in all of the samples. The patients included in this study had features that might have influenced the development of interstitial lung diseases and pulmonary fibrosis, such as age and certain comorbidities (Table 1) [64, 83]. However, none of these patients showed evidence of a previous pulmonary fibrosis disorder, and no fibrotic-like radiographic abnormalities were found when they were first diagnosed. These observations point to SARS-CoV-2 infection as a driver of the observed pathological changes, which were possibly enhanced by age and comorbidities. The ability of SARS-CoV-2 to induce fibrosis was also shown in infected aged hamsters. Interestingly, although there was evidence of proliferating AT2, the abundant loss of alveolar epithelial cells suggests that the epithelial cell regenerative response failed to restore the damaged alveolar epithelium. This is consistent with other studies describing impaired AT2 regeneration in postmortem lungs from COVID-19 cases [59, 74].

Detection of NC in lung samples from patients R, J, D, and B indicated the presence of SARS-CoV-2 at advanced stages of DAD. Thus, the approach used in this work to detect NC in human tissues, based on indirect immunofluorescence (IF) followed by confocal microscopy analysis, was found to be effective for monitoring the presence of SARS-CoV-2 in a variety of samples, in agreement with our previous report [25]. In addition, postmortem tissue samples were collected soon after the patient’s death, thus limiting tissue damage and increasing the chances of detecting both viral and host antigens under our experimental conditions. Although a small number of cases were studied in this work, our IF approach performed similarly to the highly sensitive approaches used by others, such as RT-qPCR and RNA sequencing, for detection of SARS-CoV-2 at advanced stages of DAD [21, 68, 75, 110]. On the other hand, IF has been described to be more sensitive and specific than immunohistochemistry (IHC) [45, 109] and has been used successfully by others to detect SARS-CoV-2 in different tissue samples, including kidney and liver [68, 108]. Accordingly, some reports have illustrated that IHC could be less specific than *in situ* hybridization (ISH) to detect SARS-CoV-2 in formalin-fixed paraffin-embedded (FFPE) tissue samples [58, 110].

The presence of NC in the interface of the capillary endothelium and alveolar epithelial cells as well as adjacent connective tissue suggests that SARS-CoV-2 may contribute directly to sustained damage and interference with the alveolar air-blood interface, deregulation of the wound-healing response (WHR) and immune responses leading to impaired viral clearance, reduced epithelium regeneration, tissue remodeling, and pathology. In addition, detection of NC in ELCs and their associated damage, not only in lung samples but also in kidney and liver, strongly suggests viral infection of endothelial cells. This is in agreement with other studies describing the presence of SARS-CoV-2 components in lung capillary endothelium and increased ACE2 expression in activated vascular endothelium [21, 42, 49, 56, 78, 102, 113, 118]. Macrophages have been shown to be key players during SARS-CoV-2 infection and its associated pathogenesis [25, 34, 59, 74, 118]. Interestingly, NC was identified in CD163^+^ and CD68^+^ cells corresponding to alveolar and interstitial MLCs, in accordance with other reports [14, 21, 25, 74, 105]. Importantly, co-detection of NC with NLRP3 and IL1β in MLCs and possibly in LPFs (see below) might indicate direct viral induction of inflammatory responses, which has been associated with COVID-19 pathogenesis [39]. We observed the common occurrence of mitochondrial damage in different cells in the tissues analyzed by electron microscopy. Colocalization of NC with PHB and PGC1α suggests that NC could be recruited to or close to mitochondria, suggesting a possible direct virus-mediated mitochondrial dysfunction, which may be associated with the generation of mtROS and mtDNA, contributing to NLRP3 activation and production of IL1β. Evidence supporting this view includes the observations that SARS-CoV-2 infection affects mitochondria structure and function [17], NSP2 interacts with mitochondrial PHB [20], and viral double-stranded RNA (dsRNA) is localized in mitochondria, leading to mitochondrial dysfunction in infected cultured cells [84]. Thus, mitochondrial dysfunction mediated by both viral and inflammatory responses may be connected to the ability of SARS-CoV-2 infection to stimulate the NLRP3 inflammasome and IL1β production [25, 95].

Lung fibrosis have been suggested to contribute to the progression of COVID-19 disease and post-COVID-19 sequelae [74, 103]. Notably, NC was detected in connective tissue and FLCs. LPFs are adipocyte-like cells that play a key role in mesenchymal-epithelial communication, providing triglyceride substrate to AT2 for surfactant synthesis [72]. Of note, impairment of homeostatic communications between AT2 and LPFs in the alveolar wall, leading to surfactant insufficiency, has been implicated in chronic lung diseases. These communications play an essential role in the repair response to lung injury, supporting AT2 growth and differentiation [72]. A key feature of this process is the activation of PPARγ signaling in LPFs induced by AT2-produced parathyroid hormone-related protein. Results from this work suggested the presence of SARS-CoV-2 in LPFs. The lipogenic nature of these cells was suggested by the concomitant detection of LDs and activated PPARγ. Thus, SARS-CoV-2 may impact LPFs, disrupting normal mesenchymal-epithelial homeostatic communications and surfactant production and contribute to lung pathogenesis. This may be particularly relevant, as reduced pulmonary surfactant levels are a hallmark of COVID-19 ARDS [81]. Impaired regulatory functions of LPFs may promote transdifferentiation to myofibroblasts and increased fibrosis. This is also important given that transdifferentiated LPFs are unable to support AT2 growth and differentiation during injury/repair responses [97]. Therefore, together with the direct influence of SARS-CoV-2 on AT2, this study raises the interesting possibility that SARS-CoV-2 may disrupt the regulatory functions of LPFs to promote fibrosis and disturb epithelial regeneration. Interestingly, PPARγ agonists have been used to promote repair responses in the lung by restoring epithelial–mesenchymal interactions and alveolar homeostasis in various models of lung injury [72]. Collectively, these observations point to PPARγ as a potential therapeutic target for COVID-19.

Additional findings from this work indicating the occurrence of SARS-CoV-2 in fibronectin^+^ FLCs support the above-mentioned hypothesis. Fibronectin is a key component of the ECM involved in the pathogenesis of lung diseases. Although collagens are the predominant ECM proteins identified in fibrotic lesions, highly increased levels of fibronectins have been described to localize in pulmonary areas of active fibrogenesis [51]. Consequently, increased fibronectin deposition and fibronectin expression in fibroblasts have been described in various pathological conditions of the lung, including idiopathic pulmonary fibrosis (IPF), COPD, and cancer [37, 50, 62, 120]. Fibronectin has also been described to induce EMT of alveolar epithelial cells during lung injury, a cellular process that is involved in the opening of epithelial barriers and cell migration [47]. Thus, co-detection of NC with fibronectin in the connective tissue and FLCs suggest that SARS-CoV-2 may modulate fibronectin production and related functions [98]. Inhibition of fibronectin assembly has been proposed as a therapeutic opportunity for fibrosis [2, 98] and may also be considered as a potential target against SARS-CoV-2-related lung pathology.

VMT, on the other hand, has been implicated in IPF and the invasive properties of fibroblasts in IPF, EMT during pulmonary fibrosis, non-alcoholic steatohepatitis, and hepatocellular carcinoma [47, 53, 76, 94, 107, 117]. The results from this study are in accordance with a recent report describing colocalization of VMT and the SARS-CoV-2 M protein in fibrotic lungs from COVID 19 patients [101]. However, although the role of VMT in lung WHR has not been completely elucidated, it has been shown to be required for remodeling of the alveolar epithelium and increased wound repair [47, 76]. Thus, usurping key VMT functions by SARS-CoV-2 in alveolar epithelial cells may contribute to impaired epithelial regeneration and WHR. Conversely, as VMT is involved in the life cycle of several viruses, including HIV, SARS-CoV, and SARS-CoV-2, it is considered to be an important antiviral target [28, 69, 115]. VMT has been shown to be required for SARS-CoV-2 replication and entry [3, 17, 93]. Accordingly, an interesting possibility is that EMT and increased VMT expression may render cells more susceptible to SARS-CoV-2 infection, particularly by facilitating viral entry and replication. Notably, a therapeutic peptide that modifies the supramolecular structure of VMT intermediate filaments has been shown to inhibit infection with betacoronaviruses, including SARS-CoV-2, in cell culture [28, 29]. Additionally, VMT is involved in inflammatory and fibrosis responses in the lung through activation of the NLRP3 inflammasome and induction of IL1β [24]. In this work, we found evidence of the possible involvement of VMT-expressing and LDs-containing cells in NLRP3 responses. Thus, in addition to MLCs, other cell types (including FLCs and epithelial cells with features of EMT) might contribute to inflammasome-mediated inflammatory responses. Taking into account that IL1β has been shown to play a role in lung injury and pulmonary fibrosis [44, 48], detection of NC in VMT-positive cells (including FLCs) indicates the involvement of VMT not only in the viral life cycle but also the pathogenesis induced by SARS-CoV-2 infection.

Previous studies have shown that chronic inflammation driven by IL6 and macrophage-derived IL1β is associated with impaired alveolar regeneration through induction of damage-associated transient progenitors (DATPs) from AT2 cells that are unable to make a full transition to AT1 cells during pulmonary fibrosis in both humans and mice [18, 19]. Notably, lung fibrotic fibroblasts and immunosuppressive PD1^+^ macrophages have been linked to pulmonary fibrosis and an impaired adaptive T-cell immune response in both humans and mice [18, 36]. Similarly, increased macrophages expressing IL1β and lung fibrotic fibroblasts have been associated with DATPs in impaired alveolar regeneration during SARS-CoV-2 infection [21, 59]. Interestingly, increased proximity and interactions between MLCs and FLCs have been observed in late COVID-19 disease associated with expansion of mesenchymal cells and fibroblasts [74]. By upregulating CD47 and PDL-1, fibrotic fibroblasts enhance their survival, avoiding phagocytosis by PD-1^+^ macrophages while contributing, with IL6, to inflammation. Notably, combined immunotherapy with CD47- and IL-6-blocking agents has been shown to reverse fibrotic conditions in mice, suggesting new therapeutic alternatives for treating pulmonary fibrosis [18, 54]. Our work provides additional evidence that SARS-CoV-2 may directly influence this immunoregulatory route, thus contributing to the development of fibrosis and failure of the compensatory alveolar epithelial regeneration response.

The localization of NC to LDs indicates a link between SARS-CoV-2 and lipid metabolism and LD biogenesis. An association between SARS-CoV-2 infection and lipid metabolism and LDs has been demonstrated in cell culture and animal models as well as in virus-infected patients [22, 35, 65]. NC has been shown to induce expression of diacylglycerol acyltransferase (DGAT) and LD formation [22, 116]. Further association of NC with adipocyte differentiation-related protein (ADRP) on the surface of LDs promotes the viral replication cycle. Interestingly, interfering with LD synthesis inhibits SARS-CoV-2 replication and associated cell and pulmonary inflammation in cell culture and animal models of viral infection, implicating LDs not only in the viral life cycle but also in lung pathogenesis [22, 116]. Another interesting finding of the current work was the simultaneous localization of NC with LC3B and DDX3X, which were also detected on LDs. This suggests the possible involvement of these host factors in the viral life cycle, pointing to LDs as a platform involved not only in inflammation, viral replication, and morphogenesis but also in the regulation of cellular functions and processes associated with these proteins (see Supplementary Discussion).

### Findings in postmortem kidney and liver

The results of this work pointed to the ability of SARS-CoV-2 to infect various cell types from kidney and liver. This supports previous reports of SARS-CoV-2 in several organs, including kidney and liver [11, 68, 108]. Lung-kidney interactions during SARS-CoV-2 infection are common and associated with significant morbidity and mortality [63]. Importantly, the presence of SARS-CoV-2 in the kidney has been associated with older age, an increased number of coexisting conditions, acute kidney injury, and increased risk of premature death within the first 3 weeks of disease [12]. These features were present in the cases examined in this work. We recognize that pathological features observed in the studied cases, particularly interstitial fibrosis and frequent occurrence of LDs, could be related to the combination of age and co-morbidities in these patients (Table 1) [43, 60, 96]. However, SARS-CoV-2 infection and direct viral injury could also contribute to renal tissue damage. SARS-CoV-2 infection of tubular epithelial cells has been shown previously to be associated with acute tubular renal injury [1].

The detection of SARS-CoV-2 in a variety of renal cell types such as epithelial tubular cells, endothelial cells, glomerular podocytes, and mesangial cells is consistent with the previously reported wide cellular tropism of SARS-CoV-2 in kidney [68]. Another interesting finding was the presence of NC in both cortical peritubular and medullary fibronectin^+^ interstitial cells. In accordance with the findings in lung and liver (see below), NC was co-detected with LDs that also co-stained with VMT, LC3B, and DDX3X. FLCs form the major mass of interstitial cells and perform a variety of endocrine functions in different intrarenal zones [52]. It is interesting to note that localization of NC in interstitial cells was associated with detection of collagen-like fibers in the renal interstitium. Interstitial cells from the peritubular capillary bed of the renal cortex have been involved in sensing the arterial oxygen content, which is related to alveolar oxygen tension and alveolar gas exchange [31, 46]. This process regulates the production of erythropoietin (EPO) and erythropoiesis by renal interstitial cells [52]. Consequently, hypoxemia due to SARS-CoV-2-infection-associated lung disease is a key trigger of EPO production. Thus, the presence of SARS-CoV-2 in peritubular cells of the renal cortex may also contribute to disturbance of the normal regulation of oxygen homeostasis, thus contributing to the pathogenesis of COVID-19.

Notably, in some glomerular regions, NC localized predominantly to the juxtaglomerular apparatus, including epithelial cells of the macula densa, juxtaglomerular/perivascular interstitial cells, and extraglomerular mesangial cells. This finding raises the possibility that SARS-CoV-2 may affect and deregulate critical functions of these cells such as regulation of the renin-angiotensin-aldosterone system (RAAS), which is involved in blood pressure regulation and electrolyte homeostasis [80]. Importantly, the tissue balance between ACE and ACE2 activity regulates the effector functions of RAAS, including inflammatory and fibrotic responses [33]. It has been proposed that SARS-CoV-2 infection may diminish the effects of ACE2, favoring ACE-related functions [86]. We would like to propose that SARS-CoV-2 interactions with FLCs and the juxtaglomerular apparatus directly promote pro-inflammatory and pro-fibrotic responses in the lungs and kidneys, thus contributing to RAAS imbalance. We propose that this scenario is particularly relevant in individuals who have various concomitant co-morbidities and, consequently, are at increased risk of infection of the kidney by SARS-CoV-2, contributing to premature death [12]. Further studies are needed to understand the functional implications of SARS-CoV-2 infection of these cells for regulation of RAAS and oxygen homeostasis and viral pathogenesis.

COVID-19 severity has been associated with acute liver injury and elevated liver enzymes [85, 108], and various mechanisms have been proposed [32]. On the other hand, SARS-CoV-2 has been detected in postmortem liver samples [8, 68, 70, 108]. In this work, we identified NC in hepatocytes and CD34^+^ cells, possibly representing SLECs. CD34 may be expressed at low levels in SLECs in the normal liver, depending on zonation, which increases under pathological conditions [66, 91]. Accordingly, CD34 has been associated with capillarization of LSECs in a mouse model of cirrhosis [91]. Therefore, the presence of SARS-CoV-2 in liver CD34^+^ cells may be associated with viral pathogenesis and/or pre-existing conditions in the patient, such as hepato-steatosis (which was the main pathological finding in liver samples). Interestingly, NC co-localized with VMT showing a characteristic granulated pattern, suggesting altered VMT localization and its involvement in viral life cycle. The abundance of LDs in the liver samples made it easier to study the recruitment of NC, VMT, DDX3X, LC3B, and NLRP3 to or near LDs. VMT has been shown to play a critical role in LD biogenesis [40], regulation of autophagy [9, 106], and NLRP3 inflammasome activation [24]. Concomitant detection of SARS-CoV-2, VMT, LDs, LC3B, and NLRP3 supports the role of VMT and LDs in the viral life cycle and pathogenesis involving autophagy and inflammasome functions. This is also particularly relevant because VMT has been observed in injured hepatocytes and may be associated with the pathogenesis of liver diseases [53, 112, 117] and de-regulated inflammatory responses [24]. In particular, it has been shown that an EMT-like phenotype and expression of EMT markers such as VMT are induced under various pathological conditions in the liver, including steatohepatitis and fibrosis in humans and mice [53, 92, 119]. It is therefore possible that previous pathological conditions and/or injury of the liver caused by viral infection may promote VMT expression and infection of hepatocytes by SARS-CoV-2.

## Conclusions

We have identified potential cellular and molecular targets that may be related to and affected by SARS-CoV-2 infection, with implications for virus-induced pathogenesis and therapeutics. This study provides evidence for the presence of SARS-CoV-2 in lung epithelium, MLCs, FLCs, and LPFs at advanced stages of DAD development, suggesting sustained viral injury and deregulation of tissue repair functions; NC colocalization with mitochondrial proteins and frequent mitochondrial damage in analyzed samples, pointing to mitochondrial involvement in the viral life cycle and pathogenesis; SARS-CoV-2-associated NLRP3 and IL1β responses related to VMT and LDs, not only in MLCs but also in FLCs, possibly associated with mitochondrial dysfunction; the presence of NC in regulatory cells expressing immune-checkpoint proteins involved in tissue repair responses and contributing to inflammatory responses in the lung; key host proteins localizing with NC and/or LDs that have been implicated in WHR and/or the SARS-CoV-2 life cycle (VMT, NLRP3, LC3B, DDX3X, fibronectin, and PPARγ); the presence of SARS-CoV-2 in endothelial cells from lungs, kidney, and liver, which is probably involved in endothelial damage and tissue injury; the presence of SARS-CoV-2 in hepatocytes expressing vimentin, renal interstitial cells, and the juxtaglomerular apparatus, suggesting possible virus-mediated deregulation of critical hepatic and renal functions involved in RAAS, oxygen tension regulation, and COVID-19 pathogenesis.

## Supplementary Information

Below is the link to the electronic supplementary material.

Supplementary file1 (DOCX 54 KB)

Supplementary file2 (TIF 20487 KB)

Supplementary file3 (TIF 8936 KB)

Supplementary file4 (TIF 7876 KB)

Supplementary file5 (TIF 8229 KB)

Supplementary file6 (TIF 6929 KB)

Supplementary file7 (TIF 7671 KB)

Supplementary file8 (TIF 1263 KB)

Supplementary file9 (TIF 1165 KB)

Supplementary file10 (TIF 1223 KB)

Supplementary file11 (TIF 644 KB)

Supplementary file12 (TIF 1235 KB)

Supplementary file13 (TIF 1254 KB)

Supplementary file14 (TIF 642 KB)

Supplementary file15 (JPG 6770 KB)

Supplementary file16 (TIF 6423 KB)

Supplementary file17 (TIF 1083 KB)

Supplementary file18 (TIF 1226 KB)

Supplementary file19 (TIF 3955 KB)

Supplementary file20 (TIF 7538 KB)

Supplementary file21 (TIF 639 KB)

Supplementary file22 (TIF 1114 KB)

Supplementary file23 (TIF 1338 KB)

## Data Availability

Data sharing is not applicable to this article, as no datasets were generated or analyzed during the current study.
