# Supplementary material for: Evidence of SARS-CoV-2 infection in postmortem lung, kidney, and liver samples, revealing cellular targets involved in COVID-19 pathogenesis
Source: Arch Virol. 2023 Feb 26;168(3):96. doi: 10.1007/s00705-023-05711-y (PMC9968404; doi:10.1007/s00705-023-05711-y)
Supplement: Supplementary file 1 — Supplementary file1 (DOCX 54 KB) [file 705_2023_5711_MOESM1_ESM.docx]

**Supplementary Information (SI)**

**Title:** Evidences of SARS-CoV-2 infection in postmortem lung, kidney and liver samples revealing cellular targets involved in COVID-19 pathogenesis

**Journal**: Archives of Virology

**Authors**

Viviana Falcón-Cama^a,j♦*^, Teresita Montero-González^b♦*^, Emilio F. Acosta-Medina^c,j*^, Gerardo Guillen-Nieto^a,j^, Jorge Berlanga-Acosta^a,j^, Celia Fernández-Ortega^a,j^, Anabel Alfonso-Falcón^d^, Nathalie Gilva-Rodríguez^a^, Lilianne López-Nocedo^a^, Daina Cremata-García^a^, Mariuska Matos-Terrero^a^, Giselle Pentón-Rol^a,j^, Iris Valdés^a^, Leonardo Oramas-Díaz^a^, Anamarys Suarez-Batista^e^, Enrique Noa-Romero^e^, Otto Cruz-Sui^e^, Daisy Sánchez^c^, Amanda I. Borrego-Díaz^c^, Juan E. Valdés-Carreras^c^, Ananayla Vizcaino^c^, José Suárez-Alba^a^, Rodolfo Valdés-Véliz^a^, Gretchen Bergado^f^, Miguel A. González^f^, Tays Hernandez^f^, Rydell Alvarez-Arzola^f^, Anna C. Ramírez-Suárez^a^, Dionne Casillas-Casanova^a^, Gilda Lemos-Pérez^a^, Omar R. Blanco-Aguila^g^, Angelina Díaz^c^, Yorexis González^c^, Mónica Bequet-Romero^a^, Javier Marín-Prida^h^, Julio C. Hernández-Perera^d^, Leticia del Rosario-Cruz^b^, Alina P. Marin-Díaz^i^, Maritza Gonzalez-Bravo^j^, Israel Borrajero^k^, Nelson Acosta-Rivero^l,m♦*^

a: Center for Genetic Engineering and Biotechnology (CIGB), Habana, Cuba.

b: Hospital ´Luis Díaz Soto´, Habana, Cuba

c: Center for Advanced Studies of Cuba, Habana, Cuba

d: Hospital and Center for Clinic and Chirurgic research, Habana, Cuba

e: Department of Virology, Civilian Defense Scientific Research Center, Mayabeque, Cuba

f: Direction of Immunology and Immunotherapy, Center of Molecular Immunology, Habana, Cuba.

g: CIGB, Sancti Spiritus, Cuba

h: Center for Research and Biological Evaluations, Institute of Pharmacy and Food, University of Havana, Havana, Cuba.

i: International Orthopedic Scientific Complex ´Frank Pais Garcia´, Habana, Cuba

j: Latin American School of Medicine, CallePanamericana Km 3 1/2, Playa Havana, 11600, Cuba.

k: Clinical Surgical Hospital ´Hermanos Ameijeiras´, Habana, Cuba

l: Center for Protein Studies, Department of Biochemistry, Faculty of Biology, University of Habana, Habana, Cuba.

m: Centre for Integrative Infectious Disease Research (CIID), Molecular Virology, University of Heidelberg, D-69120, Heidelberg, Germany.

♦These authors contributed equally to this study and should be considered as first authors

*Corresponding authors:

Prof. Viviana Falcón-Cama, PhD.

-Center for Genetic Engineering and Biotechnology (CIGB), Ave 31 be/ 158 and 190, Cubanacán, Playa, PO Box 6162, Havana 10699. Cuba. E-mail: viviana.falcon@cigb.edu.cu

Prof. Teresita Montero González, PhD.

-Hospital "Luis Díaz Soto". La Habana, Cuba. E-mail: teremg@infomed.sld.cu

Prof. Emilio F. Acosta-Medina, PhD.

-Center for Advanced Studies of Cuba, and Latin American School of Medicine, CallePanamericana Km 3 1/2, Playa, Havana 11600, Cuba. emilio.am@cea.cu

Dr, Nelson Acosta-Rivero, PhD.

-Center of Protein Studies, Department of Biochemistry, Faculty of Biology, University of Habana, Calle 25 entre J e I, #455, Plaza de la Revolucion, Habana, 10400. Cuba. E-mail: nelson.acosta@fbio.uh.cu

-University of Heidelberg, Medical Faculty Heidelberg, Department of Infectious Diseases, Molecular Virology, Centre for Integrative Infectious Disease Research (CIID), INF 344, GO.1, D-69120 Heidelberg, Germany. nelson.acosta@med.uni-heidelberg.de

**Supplementary Methods**

**Transmission Electron Microscopy**

Transmission electron microscopy (TEM) of collected cells and tissue samples was performed as previously described with minor modifications (1, 2). Briefly, collected samples were first fixed in 3.2 % glutaraldehyde diluted in 0.1M sodium cacodylate (pH 7.4) for 1 hour at room temperature and then overnight in Cacodilate Buffer pH 7.2 at 4 °C. Afterwards, samples were post-fixed in 1% osmium tetroxide for 1 hour and dehydrated in increasing concentrations of ethanol and then embedded in Spurr (Sigma-Aldrich Co., USA) as previously described (1). Ultrathin sections (400–500 A) made with an ultramicrotome (NOVA, LKB) were placed on 400 mesh grids, stained with saturated uranyl acetate and lead citrate, and examined with a MIRA3-TESCAN Scanning Electron Microscope (TESCAN, Czech Republic) at 25.0 kV using a transmission electron detector.

**Virus propagation**

Vero E6 cells were used for isolation and initial pass as previously described (2). Briefly, Vero E6 monolayers were inoculated (ATCC CRL-1586), in 24-well plate, with two- hundred μL of clinical specimens and cultured at 37°C in 5% carbon dioxide atmosphere. After 6 days, cultures were freezed (-85°C) and thawed (37°C), cells were scraped from the well and 100 μL of virus lysates were used to inoculate 24-well plates with the Vero E6 cell line. After 3 days, cells´ monolayers were scrapped, and cultures of infected Vero E6 cells were centrifuged and fixed as shown above for transmission electron microscopy analysis. Supernatants were aliquoted and frozen for RT-qPCR analysis with specific primers and probes against SARS-CoV-2.

**SARS-Cov-2 infection of Hamsters**

Infection of Hamsters (obtained from the Center for Laboratory Animal Production (CENPALAB; Cuba) with SARS-Cov-2 was done as previously shown with minor modifications (3). In brief, two female hamsters, age (>20 weeks), 90g weight, were intraperitoneally anaesthetized by 2.5% avertin with 0.02 ml/g body weight, and then intra-nasally inoculated with 10^2^ TCID_50_ of isolated SARS-CoV-2 stock virus from patient R (2). Two hamsters intranasally inoculated with an equal volume of PBS were used as a mock-infection control. After 6 days, animals were dissected to collect lungs to detect SARS-CoV-2 through RT-qPCR and immunofluorescence analysis, and for pathological checking by TEM. These studies were performed in an animal biosafety level 3 (ABSL3) facility using HEPA-filtered isolators. All procedures were reviewed and approved by the Institutional Animal Care and Use ethics Committee from the CIGB. All animal studies complied with the ARRIVE guidelines.

**RNA extraction and quantitative RT-qPCR**

Viral RNA extraction from cell culture media was performed from 140 μL of sample according to the instructions for use of the QIAamp Viral RNA MiniKit kit (QIAGEN, Valencia, CA, USA). Lung homogenates (1 g/ml) from Hamsters were prepared by homogenizing perfused tissues in DMEM. The homogenates were centrifuged at 3,000 rpm for 10 min at 4°C and supernatants were collected and stored at −80°C. Total RNA was extracted from lung homogenates using the RNeasy Mini Kit (Qiagen). The extracted RNAs were immediately used as template in reverse- transcription-quantitative real-time PCR (RT-qPCR) as previously described for SARS-CoV-2 (4) through detection of the gene E of SARS-CoV-2 envelope protein. The Super ScriptTM III PlatinumTM One Step qRT-PCR Kit (Invitrogen) and the Rotor Gene Q-5 plex real-time PCR platform (QIAGEN) were used. Cycling conditions were: 50°C-30 min, 55°C, 95°C 2 min, 45 cycles: 95°C 15 sec, 60°C 30 sec.

**Colocalization Analysis**

Colocalization between the different channels was analyzed from image stacks using the open source image processing package Fiji (National Institute of Health) with Just Another Colocalization Plugin (JACoP) (5). Z-projections were done from z-stack series using Fiji software. Then, region of interests (Rois) were selected and analyzed with JACoP. Various global statistic approaches performing intensity correlation coefficient-based analyses offering complementary information were used. Pearson’s correlation coefficient (PC) describes the spread of the pixel distribution within a scatter plot in respect to the fitted line displaying the relationship between the intensities of the two fluorochromes. Its value can range from 1 to −1, with 1 standing for complete positive correlation and −1 for a negative correlation, with zero standing for no correlation. The Costes´s approach provides statistical significance to the calculated PC, which is evaluated after the images are thresholded to remove background fluorescence. It also compares randomized (1000 randomization rounds were used) with original images followed by evaluation of the significance (P-value) of the PC, which is the probability that true colocalization (P-value>0.95%) is present in a selected region of the image (6). Manders’ overlap coefficient, is based on the PC with average intensity values being taken out of the mathematical expression (7). Pixels from image A (FITC channel, for example) are considered colocalized, if their intensity in channel B (A647, for example) is above 0 (M1), and vice versa (M2). Values range from 0 to 1 for non- and complete overlapping events. Thus, M1 and M2 represent the fraction of one fluorescent channel overlapping the other one (M1: FITC channel overlapping A647 channel; M2: A647 channel overlapping FITC channel).

Li´s scatter plots are interpretable representations of colocalization to discriminate coincidental events in heterogeneous situations (8). The intensity correlation analysis results are presented in a set of two graphs where the x-value is dependent on covariance of both channels and the y-value reflects the normalized intensity distribution of the current channel. Pixels with values situated left of the x = 0 line do not colocalize or have inversely correlated intensities, whereas pixels situated on the right side adopting a C shape colocalize. In addition, the intensity correlation quotient (ICQ) varies from colocalization with 0.5 to exclusion with –0.5, whereas random staining and images corrupted by noise give values close to 0.

The van Steensel´s cross-correlation analysis involves shifting the green image in the x-direction pixel per pixel relative to the red image and calculating the respective PC (9). PCs are then plotted as the function of dx (pixel shift; with a pixel shift of d=±20 in this case) thus obtaining a cross-correlation function (CCF). Completely colocalizing structures peak at dx=0 and show a bell-shaped curve. Good signal to noise ratio produce CCF close to 1, although a difference in fluorescence intensity or noise leads to a reduction of the height of the bell-shaped curve whereas the peak is still at dx=0; in case of exclusion, it reaches a minimum at this position. If the maximum is shifted from dx=0, partial colocalization can be determined.

**Supplementary Discussion**

DDX3X is a multifunctional protein involved in several cellular processes, cancer, HCV replication and pathogenesis (10-13). DDX3X has been shown to promote the replication of several human viruses which have been shown to counteract DDX3X-mediated innate immunity and stress granules formation (14-16). Importantly, DDX3X has been shown as a valuable anti-viral and anti-cancer target using small molecular inhibitors of DDX3X (14, 17). DDX3X colocalized with SARS-CoV-2 NC and LDs suggesting its involvement in viral life cycle linked with trafficking to LDs. This is analogous to previous observations in HCV infected cells (10). Our findings also support other reports showing that DDX3X interacts with NC in SARS-CoV-2-infected cells (18), and that it is a host target for SARS-CoV-2 infection (19) and antiviral therapy (20). Additional studies are needed to define the role of DDX3X during SARS-CoV-2 infection.

LC3B is a key host factor involved in autophagy, especially in substrate uptake upon binding to several autophagy receptors, autophagosome maturation and autolysosome formation (21). Importantly, autophagy is involved in the initial control of viral infection, antigen presentation and the regulation of excessive inflammatory response such as that triggered by the inflammasome activity (22, 23). Autophagy has been involved in the life cycle of coronaviruses promoting the formation of double-membrane vesicles (DMVs) where viruses replicate (24, 25). SARS-CoV-2 infection has been described to inhibit autophagy flux by blocking fusion of autophagosomes with lysosomes resulting in accumulation of autophagosomes (26). Autophagosomes, on the other hand, are required for SARS-CoV-2 release (27). Interestingly, LDs may promote macroautophagy contributing to autophagosome biogenesis (28, 29). On the other hand, autophagy inhibition may affect the control of inflammatory responses, particularly those related to inflammasomes´ activation contributing to viral-induced pathogenesis. Whether NC and LC3B colocalization are related to either autophagy inhibition, viral release and/or other relevant viral-cellular interactions require further studies. However, recruitment of LC3B to LDs during viral infection also suggests the interesting possibility that SARS-CoV-2 could modulate lipophagy. Inhibiting lipophagy may, on one hand, increase accumulation of LDs to promote LDs-related viral replication while inducing lipophagy may, on the other hand, inhibit antiviral effects and provide a key source of energy (ATP generated by β-oxidation in mitochondria) for viral life cycle as has been shown for other viruses (30). It is interesting to note that flavivirus proteins have been involved in transferring regulatory proteins from LDs to autophagosomes to induce lipophagy (31). Further studies on this topic will contribute to elucidate the relevance of viral and host factors recruitment to LDs and their relationship to autophagy/lipophagy during SARS-CoV-2 infection.

**Legends to supplementary figures**

**-Supplementary Fig 1 (Fig S1)**

# Representative images of Masson's trichrome staining

**1**: Quantification of Masson's trichrome staining (MTS) in lung sections of cases studied. Results shown as the ratio of MTS´ area/Interstitium area. **2**: Lung sections of patient R showing: **A**) interstitial fibrosis (blue) with features of fibrosis by accretion (arrows), **B**) alveolar damage with ruptured intra-alveolar septa and destruction of the alveolar wall lining with sloughing and desquamation of pneumocytes (arrowheads) leaving a denuded basement membrane (curved arrow); alveolar type 2-like pneumocytes (AT2) (arrows) proliferating along the surface of fibrous alveolar septa; **C)** thickening of alveolar septa by proliferating fibroblasts and deposition of collagen (arrow). Also note an alveolar bud of fibrous tissue at the tip of the alveolar septa covered by hyalin-like membrane (arrowhead); **D**) Part of alveolar duct containing hyaline membranes layering on alveolar septa (arrows, stained pastel purple) and abundant collagen fibers (blue). Note a fibrous bud and fibrosis by accretion near hyaline membranes (arrowhead); **E**) loss of the alveolar epithelial barrier and interstitial fluid escape into the alveolar space (arrows). Note proteinaceous and cellular alveolar exudate; **3**: Lung sections of patients **T**, **J**, **D** and **B** showing interstitial fibrosis. **Bars**: 50 µm. (Original magnifications ×40). Collagen is stained in blue, hyaline membranes in pastel purple, erythrocytes in orange-yellow, keratins and cell cytoplasm in red and nuclei dark brown/black.

**-Supplementary Fig 2 (Fig S2)**

Transmission electron micrographs showing ultrastructural features in lung sections of patient R (S2A) and in lung sections of aged hamsters infected or not with SARS-CoV-2 isolated from patient R (S2B).

**S2A: A**) Cell debris (arrowheads) in alveolar space (AS), detachment of damaged epithelial cell (arrows) leaving a denuded alveolar epithelial basal lamina (curved arrow). **B**) Abundant connective tissue fibers including collagen-like fibers (CF) and a Fibroblast-like cell (F) in the alveolar interstitium. **C**) Proliferating alveolar type II pneumocytes (AT2). **D**) An alveolar type II pneumocyte (AT2) lying free in the AS. **E,F**) Part of a lipofibroblast-like cell showing lipid droplets (LD), dilated endoplasmic reticulum (ER) and damage mitochondria (M) (**E**) and presence of large LD (**F**) in the alveolar interstitium. Note denuded alveolar epithelial basal lamina (curved arrows). **Bars**: 1 µm. Capillaries (C); Erythrocyte (E); alveolar interstitium (i); nucleus (N); lamellar-like body (*), lysosome (Ly).

**S2B:** Part of lung sections from a mock-infected control **A)** showing normal alveolar septa and from a SARS-CoV-2 infected hamster **B)** illustrating thickened alveolar septa due to interstitial edema and accumulation of connective tissue fibers (CF). Also note CF in the alveolar space (AS). **Bars**: 1 µm.

**-Supplementary Fig 3 (Fig S3)**

Electron microscopy analysis of Vero E6 cells infected with SARS-CoV-2 isolated from patient R.

A) Part of uninfected Vero E6 cell. B) Vero E6 cells infected with SARS-CoV-2 (72 hours post-infection); Ba: Part of a Vero E6 cell showing virus-like particles in intracellular vesicles and in close contact with the cell membrane (arrowheads); Bb: cell debris containing virus-like particles (arrowheads); Note below, range of Ct values for E gene in these samples (ND: No detection). **Bars**: 1 μm.

**-Supplementary Fig 4 (Fig S4)**

**Detection of SARS-Cov-2 nucleocapsid (NC) in tissue samples from SARS-Cov-2-infected Hamsters and deceased COVID-19 patients**

**A, B, C**) Lung sections from SARS-Cov-2-infected (upper panels) and mock-infected (lower panels) Hamsters, incubated with different antibodies against NC (mouse monoclonal IgG antibodies, SINO Biologicals (**A**) and CIGB Sancti Espiritus (**B);** rabbit polyclonal antibodies (**C**) followed by Alexa 647 (A647)-conjugated anti-mouse/rabbit IgGs, and DAPI to stain nucleus (blue channel); arrows indicate NC staining observed only in lung sections from SARS-Cov-2-infected animals but not from mock-infected ones; Note on the right, range of Ct values for E gene in these samples (ND: No detection); 10X magnifications; **Bars:** 100 µm. **D**) Lung section from a person who died from a cause non-related to COVID-19 showing no staining for NC (anti-NC rabbit polyclonal antibodies followed by A647-conjugated anti-rabbit IgGs). As a reference, a mouse monoclonal antibody against the microtubule associated protein MAP2 was used (followed by fluorescein-conjugated anti-rabbit IgGs) (arrows), 20X magnification. **E)** Lung sections from patients J, D and B incubated with rabbit polyclonal anti-NC (followed by A647-conjugated anti-rabbit IgGs). Note NC staining in all analyzed samples; 20X magnification. **F)** Representative images from confocal microscopy analysis of lung (left panel), kidney (middle panel) and liver (right panel) sections from patient R incubated with anti-NC mouse monoclonal IgG antibody (SINO Biologicals) (followed by either fluorescein- or A647-conjugated anti-mouse IgGs) showing the presence of NC in these samples (arrows), 40X magnifications. **Bars:** 50 µm.

**-Supplementary Fig 5 (Fig S5)**

Representative images from confocal microscopy analysis of lung sections from patients T and R, incubated with various combinations of rabbit and mouse antibodies against NC (CIGB, Sancti Espiritus) and host proteins, followed by Alexa 647 (A647)- and fluorescein/FITC-conjugated anti-mouse/rabbit IgGs either alone or in different combinations; also mouse anti-CD163 IgGs-conjugated to FITC were used; and DAPI to stain nucleus (blue channel). **Bars:** 50 µm.

**A**) Lung section of patient T showing no staining for NC (anti-NC rabbit polyclonal antibodies; A647). As a reference, a mouse monoclonal antibody against the microtubule associated protein MAP2 was used (FITC). Note a negative control of a section incubated only with secondary fluorescent probes-conjugated antibodies without primary antibodies (Merge, No MAP2, No NC); 20X magnification. **B-D_NC1)** Illustrative region of interests (Rois) of lung sections from patient R showing that: NC (A647) localized to: ACE2^+^ cells (FITC) (arrows) (**B**); CD163^+^ alveolar macrophage-like cells (MLCs) (arrowhead), as well as interstitial MLCs (arrows) (FITC) (**C**), 40X magnifications; NC (A647) was detected in the interface of the capillary endothelium and alveolar epithelial cells (arrow) and adjacent connective tissue (arrowhead), highlighted in the augmented inset with differential interference contrast optics (DIC), capillary endothelial cell (E), pneumocyte (Ep) (**D-NC1**); **D-NC2**) A tracheal wall section of patient R showing augmented inset with differential interference contrast optics (DIC) highlighting that NC (A647) was localized to epithelium (white arrowhead, inset), connective tissue including fibroblast-like cells (arrow, inset); NC was also detected in sero-mucous glands (red arrowhead), 20X magnification.

**-Supplementary Figures 6-9 (Fig S6-S9)**

**Fig. S6-9:** *Colocalization analysis from Figures 1,2,3 and Supplementary Fig 5*. Colocalization was analyzed using the open source image processing package Fiji with Just Another Colocalization Plugin (JACoP) (5). This allowed the use of several global statistic approaches performing intensity correlation coefficient-based analyses. Analyses included colocalization based on calculated thresholds according to Costes’ statistical significance algorithm (**Fig. 1-4 Supplementary Fig. 5**), and the colocalization was quantified with calculated intensity correlation quotients (ICQ), Pearson’s (PC) and Manders’ coefficients (M1, M2) (**Fig. 1-4, Supplementary Fig. 5**), and statistically evaluated with P-values by Costes’. Additional evidences for colocalization were provided by the plots of Li´s intensity correlation analysis (Upper and middle panels) and the Van Steensel’s cross-correlation functions (CCFs) (lower panels).

**Fig. S6:** Colocalization of NC with LC3B (**A**), CD34 (**B**) K10 (**C**) and concomitant with IL1B and CD163 (**D**) was supported with Costes´ PC and Manders’ (M1, M2) coefficients close to 1 and P-value of 100% (not shown). In addition, scatter plots for the images show strong right skewing for NC and host proteins. In these panels (upper and middle), “A” and “B” are the intensity of analyzed fluorophores while “a” and “b” are the average of these values, respectively. For these regions, ICQ values range from 0.37 to 0.46 (**Fig 1**). These ICQ values for co-staining of matched images indicate that pairs of analyzed signals varied in close synchrony. Moreover, plots of the Van Steensel’s CCF (lower panels) gave a bell-shaped curve with its maxima located at dx =0, indicating colocalization.

**Fig. S7:** Colocalization of NC with Fib (**A**), VMT (**B**), DDX3X (**C**), and concomitant with PPARγ-P and ORO (**D**); was verified with Costes´ PC and Manders’ (M1, M2) coefficients close to 1 and P-value of 100% (not shown). In addition, scatter plots for the images show strong right skewing for the various pairs of markers analyzed. In these panels (upper and middle), “A” and “B” are the intensity of analyzed fluorophores while “a” and “b” are the average of these values, respectively. For all analyzed Rois, ICQ values range from 0.34 to 0.47 (**Fig 2**). These ICQ values for co-staining of matched images indicate that pairs of analyzed signals varied in close synchrony. Moreover, plots of the Van Steensel’s CCF (lower panels) gave bell-shaped curves with its maxima located at dx =0, indicating colocalization.

**Fig. S8:** Colocalization of NC with ACE2 (**A**), CD163 (**B**) (**Supplementary Fig 5**) and PHB (**F**) (**Fig. 3**); Colocalization of NLRP3 with CD68 (**C**) and CD163 (**D**); Colocalization of IL1B with Fib (**E**); Colocalization of VMT concomitant with ORO and NLRP3 (**G**) (**Fig 3**); were supported with Costes´ PC and Manders’ (M1, M2) coefficients close to 1 and P-value of 100% (not shown). In addition, scatter plots for the images show strong right skewing for NC and host proteins. In these panels (upper and middle), “A” and “B” are the intensity of analyzed fluorophores while “a” and “b” are the average of these values, respectively. For these regions, ICQ values range from 0.34 to 0.45 (**Supplementary Fig 5, Fig 3**). These ICQ values for co-staining of matched images indicate that pairs of analyzed signals varied in close synchrony. Moreover, plots of the Van Steensel’s CCF (lower panels) gave a bell-shaped curve with its maxima located at dx =0, indicating colocalization.

**Fig. S9:** Concomitant localization of: NC with CD163 and PD1 (**A**), NC with IL1B and PD1 (**B**), and NC with CD47 and IL6 (**E**); CD47 with PDL1 and Fib (**C**), and CD47 with PDL1 and IL6 (**D**); was verified with Costes´ PC and Manders’ (M1, M2) coefficients close to 1 and P-value of 100% (not shown). In addition, scatter plots for the images show strong right skewing for the various pairs of markers analyzed. In these panels (upper and middle), “A” and “B” are the intensity of analyzed fluorophores while “a” and “b” are the average of these values, respectively. For all analyzed Rois, ICQ values range from 0.33 to 0.47 (**Fig 4**). These ICQ values for co-staining of matched images indicate that pairs of analyzed signals varied in close synchrony. Moreover, plots of the Van Steensel’s CCF (lower panels) gave bell-shaped curves with its maxima located at dx =0, indicating colocalization.

**-Supplementary Fig 10 (Fig. S10)**

**Fig. S10:** **A)** Representative images of Picro Mallory staining (PMS) in kidney samples of patients who died with Covid-19. PMS of postmortem renal samples from patients **R**, **J**, **D** and **B**. Kidney sections showing interstitial fibrosis. (Original magnification ×20). Bars: 200 µm. Collagen is stained in blue.

**B-D**) Representative images from confocal microscopy analysis of lung sections from patients who died with Covid-19, incubated with various combinations of mouse monoclonal antibody against NC (CIGB, Sancti Espiritus) either alone or with rabbit anti-ACE2 polyclonal antibodies, followed by Alexa 647 (A647)- and fluorescein/FITC-conjugated anti-mouse/rabbit IgGs either alone or in different combinations, and DAPI to stain nucleus (blue channel). **Bars:** 50 µm.

**B,C**) Renal cortex sections of patient R showing: NC (A647) detected in the yuxtaglomerular apparatus (arrows). Also note NC localized to interstitial cells (curved arrow). (G: glomerulus); **B2**: Tissue analyzed with DIC **(B1,2)**. NC (FITC) detection in endothelial cells (arrows), podocytes (arrowheads) and the interface of podocytes with the basal membrane surrounding arterioles (curved arrows). Note framed augmented inset analyzed with DIC; 20X magnification (**B3**). **C**) Renal cortex sections from patients J (**NC J**), D (**NC D**) and B (**NC B**). NC (A647) was detected in various regions of the yuxtaglomerular apparatus (arrows); Glomerulus (G), 20X magnifications. **D**) NC (FITC) localizing to ACE2^+^ tubule epithelial cells (A647) (arrows) and peritubular interstitial cells (arrowheads), 40X magnification.

**- Supplementary Fig 11 (Fig. S11)**

Transmission electron micrographs showing ultrastructural features in kidney sections of patient R.

**A**) Part of a proximal tubule cell from a S2 segment showing extracellular virus-like particles (VLPs) associated with plasma membrane (arrow) and VLPs in a large vesicular endomembrane compartment (black tailed arrowheads), and a damaged mitochondria (M); Nucleus (N). **Bar:** 0.5 µm**. B**) Abundant collagen-like fibers (CF) around peritubular interstitial fibroblast-like cells (F). **C**) Peritubular interstitial fibroblast-like cell (F) in the outer medulla containing lipid droplets (LD). Note capillaries (C) with an erytrocyte (E) and endothelial cells (En) . **Bars: 1 µm.**

**- Supplementary Figures 12 and 13 (Fig S12, S13)**

**Fig. S12-13:** *Colocalization analysis from Figures 5, 6 and Supplementary Fig 10.* Colocalization was analyzed using the open source image processing package Fiji with JACoP Plugin. This allowed the use of several global statistic approaches performing intensity correlation coefficient-based analyses. Analyses included colocalization based on calculated thresholds according to Costes’ statistical significance algorithm (**Fig. 5, 6 and Supplementary Fig. 10**), and the colocalization was quantified with calculated intensity correlation quotients (ICQ), Pearson’s (PC) and Manders’ coefficients (M1, M2) (**Fig 5, 6 and Supplementary Fig. 10**), and statistically evaluated with P-values by Costes’. Additional evidences for colocalization were provided by the plots of Li´s intensity correlation analysis (Upper and middle panels) and the Van Steensel’s cross-correlation functions (CCFs) (lower panels).

**Fig. S12:** Colocalization of NC with CD34 (**A**), Fib (**B**), VMT (**C**) and PHB (**D**) was supported with Costes´ PC and Manders’ (M1, M2) coefficients close to 1 and P-value of 100% (not shown). In addition, scatter plots for the images show strong right skewing for NC and host proteins. In these panels (upper and middle), “A” and “B” are the intensity of analyzed fluorophores while “a” and “b” are the average of these values, respectively. For these regions, ICQ values range from 0.41 to 0.47 (**Fig. 5)**. These ICQ values for co-staining of matched images indicate that pairs of analyzed signals varied in close synchrony. Moreover, plots of the Van Steensel’s CCF (lower panels) gave a bell-shaped curve with its maxima located at dx=0, indicating colocalization.

**Fig. S13:** Colocalization of NC with ACE2 (**B**), LC3B (**C**), DDX3X (**D**), and concomitant localization of NC with NG2 and ORO (**A**) were supported with Costes´ PC and Manders’ (M1, M2) coefficients close to 1 and P-value of 100% (not shown). In addition, scatter plots for the images show strong right skewing for NC and host proteins. In these panels (upper and middle), “A” and “B” are the intensity of analyzed fluorophores while “a” and “b” are the average of these values, respectively. For these regions, ICQ values range from 0.25 to 0.46 (**Supplementary Fig. 10, Fig.6)**. These ICQ values for co-staining of matched images indicate that pairs of analyzed signals varied in close synchrony. Moreover, plots of the Van Steensel’s CCF (lower panels) gave a bell-shaped curve with its maxima located at dx =0, indicating colocalization.

**-Supplementary Figure 14 (Fig S14)**

**Fig. S14:** Transmission electron micrographs showing ultrastructural features in hepatocytes from liver sections of patients R (A) and B (B). **A**) Part of a hepatocyte with abundant LDs and lipolysosome-related structures (arrowheads). **B**) Part of a hepatocyte showing dilated endoplasmic reticulum (ER), damaged mitochondria (M) with loss of cristae and aggregates inside, complex lipid bodies surrounded by electron-dense materials (arrowheads). Lipid droplets (LD); endoplasmic reticulum (ER); Lysosome (Lys); Nucleus (N). **Bars:** 1 µm.

**-Supplementary Figure 15 (Fig S15)**

**Fig. S15:** Representative images obtained by confocal microscopy analysis of SARS-CoV-2 NC in liver samples of patients R and B who died with Covid-19. **A**) Confocal microscopy analysis of liver sections from patients R (**NC 1, NC 2**) and B (**NC B**) incubated with mouse monoclonal anti-NC (CIGB, Sancti Espiritus) (FITC) followed by fluorescein-conjugated goat anti-mouse IgGs, and DAPI to stain nucleus (blue). Framed augmented insets analyzed with DIC. **NC 1**: Section containing a portal track (PT) region; Note that NC (FITC) localized in the connective tissue of the PT (arrows). Portal vein (PV); Hepatic artery (A); Bile duct (B). **NC 2**: Section showing NC-stained hepatocytes including NC detection in a liver sinusoidal endothelial cell (arrows); **NC B**: Section highlighting NC-stained hepatocytes. **B,C**) Liver sections from patients R and B, respectively incubated with mouse monoclonal anti-NC (CIGB, Sancti Espiritus) and rabbit policlonal anti-ACE2 antibodies; followed by either fluorescein- or Alexa 647-conjugated goat anti-mouse and either fluorescein- or Alexa 647-conjugated goat anti-rabbit IgGs, and DAPI to stain nucleus (blue). Some of NC-stained cells (FITC) colocalize with ACE2 (A647) in **A** (See Supplementary Fig 16F); Note detection of NC (A647) and scarce ACE2 staining (FITC) in **B**; **Bars**: 50 µm.

**-Supplementary Figures 16 and 17 (Fig S16, S17)**

**Fig. S16-17:** *Colocalization analysis from Figures 7, 8 and Supplementary Fig 15*. Colocalization was analyzed using the open source image processing package Fiji with JACoP Plugin. This allowed the use of several global statistic approaches performing intensity correlation coefficient-based analyses. Analyses included colocalization based on calculated thresholds according to Costes’ statistical significance algorithm (**Fig. 7, 8, Supplementary Fig 15**), and the colocalization was quantified with calculated intensity correlation quotients (ICQ), Pearson’s (PC) and Manders’ coefficients (M1, M2) (**Fig. 7, 8, Supplementary Fig 15**), and statistically evaluated with P-values by Costes’. Additional evidences for colocalization were provided by the plots of Li´s intensity correlation analysis (Upper and middle panels) and the Van Steensel’s cross-correlation functions (CCFs) (lower panels).

**Fig. S16:** Colocalization of NC with LC3B (**A**), CD34 (**B**), ORO (**C**), DDX3X (**D**), VMT (**E**) and ACE2 (**F**) was supported with Costes´ PC and Manders’ (M1, M2) coefficients close to 1 and P-value of 100% (not shown). In addition, scatter plots for the images show strong right skewing for NC and host proteins. In these panels (upper and middle), “A” and “B” are the intensity of analyzed fluorophores while “a” and “b” are the average of these values, respectively. For these regions, ICQ values range from 0.37 to 0.46 (**Fig. 7, 8A, Supplementary Fig 15**). These ICQ values for co-staining of matched images indicate that pairs of analyzed signals varied in close synchrony. Moreover, plots of the Van Steensel’s CCF (lower panels) gave a bell-shaped curve with its maxima located at dx =0, indicating colocalization.

**Fig. S17:** Concomitant localization of VMT with ORO and LC3B (**A**), with ORO and NLRP3 (**B**); was verified with Costes´ PC and Manders’ (M1, M2) coefficients close to 1 and P-value of 100% (not shown). In addition, scatter plots for the images show strong right skewing for the various pairs of markers analyzed. In these panels (upper and middle), “A” and “B” are the intensity of analyzed fluorophores while “a” and “b” are the average of these values, respectively. For all analyzed Rois, ICQ values range from 0.36 to 0.38 (**Fig 8**). These ICQ values for co-staining of matched images indicate that pairs of analyzed signals varied in close synchrony. Moreover, plots of the Van Steensel’s CCF (lower panels) gave bell-shaped curves with its maxima located at dx =0, indicating colocalization.

**References**

1. Falcon V, Acosta-Rivero N, Chinea G, Gavilondo J, de la Rosa MC, Menendez I, et al. Ultrastructural evidences of HCV infection in hepatocytes of chronically HCV-infected patients. BiochemBiophysRes Commun. 2003;305(4):1085-90.

2. Noa Romero E, Enriquez Puertas JM, Machado Zaldívar LY, González Sosa NL, Montero González TJ, Falcón Cama V, et al. SARS-CoV-2 Isolation from Cuban COVID-19 Patients. American J Rare Dis Diagn Ther. 2020;3(1):009-15.

3. Sia SF, Yan LM, Chin AWH, Fung K, Choy KT, Wong AYL, et al. Pathogenesis and transmission of SARS-CoV-2 in golden hamsters. Nature. 2020;583(7818):834-8.

4. Corman VM, Landt O, Kaiser M, Molenkamp R, Meijer A, Chu DK, et al. Detection of 2019 novel coronavirus (2019- nCoV) by real-time RT-PCR. Euro Surveill. 2020;25:2000045.

5. Bolte S, Cordelieres FP. A guided tour into subcellular colocalization analysis in light microscopy. Journal of Microscopy. 2006;224:213–32.

6. Costes SV, Daelemans D, Cho EH, Dobbin Z, Pavlakis G, Lockett S. Automatic and quantitative measurement of protein-protein colocalization in live cells. Biophys J. 2004;86:3993–4003.

7. Manders E, Visser A, Koppen A, de Leeuw W, van Liere R, Brakenhoff G, et al. Four-dimensional imaging of chromatin dynamics during the assembly of the interphase nucleus. Chromosome Res. 2003;11:537–47.

8. Li Q, Lau A, Morris TJ, Guo L, Fordyce CB, Stanley EF. A syntaxin 1, Galphao, and N-type calcium channel complex at a presynaptic nerve terminal: analysis by quantitative immunocolocalization. J Neurosci. 2004;24:4070–81.

9. Van Steensel B, van Binnendijk E, Hornsby C, van der Voort H, Krozowski Z, de Kloet E, et al. Partial colocalization of glucocorticoid and mineralocorticoid receptors in discrete compartments in nuclei of rat hippocampus neurons. J Cell Sci. 1996;109:787–92.

10. Angus AG, Dalrymple D, Boulant S, McGivern DR, Clayton RF, Scott MJ, et al. Requirement of cellular DDX3 for hepatitis C virus replication is unrelated to its interaction with the viral core protein. Journal of General Virology. 2010;91:122-32.

11. Ariumi Y, Kuroki M, Abe K, Dansako H, Ikeda M, Wakita T, et al. DDX3 DEAD-box RNA helicase is required for hepatitis C virus RNA replication Journal of Virology. 2007;81:13922-6.

12. Botlagunta M, Vesuna F, Mironchik Y, Raman A, Lisok A, Winnard P, Jr., et al. Oncogenic role of DDX3 in breast cancer biogenesis. Oncogene. 2008;27(28):3912-22.

13. Chang P-C, Chi C-W, Chau G-Y, Li F-Y, Tsai Y-H, Wu J-C, et al. DDX3, a DEAD box RNA helicase, is deregulated in hepatitis virus associated hepatocellular carcinoma and is involved in cell growth control. Oncogene. 2006;25:1991–2003.

14. Brai A, Fazi R, Tintori C, Zamperini C, Bugli F, Sanguinetti M, et al. Human DDX3 protein is a valuable target to develop broad spectrum antiviral agents. Proc Natl Acad Sci U S A. 2016;113(19):5388–93.

15. Oshiumi H, Ikeda M, Matsumoto M, Watanabe A, Takeuchi O, Akira S, et al. Hepatitis C virus core protein abrogates the DDX3 function that enhances IPS-1-mediated IFN-beta induction PLoS ONE. 2010;5:e14258.

16. Schroder M. Human DEAD-box protein 3 has multiple functions in gene regulation and cell cycle control and is a prime target for viral manipulation Biochemical Pharmacology. 2010;79:297-306.

17. Bol GM, Vesuna F, Xie M, Zeng J, Aziz K, Gandhi N, et al. Targeting DDX3 with a small molecule inhibitor for lung cancer therapy. EMBO Mol Med. 2015;7:648-69.

18. Ariumi Y. Host Cellular RNA Helicases Regulate SARS-CoV-2 Infection. J Virol. 2022;96(6):e00002-22.

19. Ciccosanti F, Di Rienzo M, Romagnoli A, Colavita F, Refolo G, Castilletti C, et al. Proteomic analysis identifies the RNA helicase DDX3X as a host target against SARS-CoV-2 infection. Antiviral Res. 2021;190:105064.

20. Vesuna F, Akhrymuk I, Smith A, Winnard Jr PT, Lin S-C, Scharpf R, et al. RK-33, a small molecule inhibitor of host RNA helicase DDX3, suppresses multiple variants of SARSCoV-2. bioRxiv preprint doi: https://doiorg/101101/20220228482334. 2022.

21. Galluzzi L, Baehrecke EH, Ballabio A, Boya P, Bravo-San Pedro JM, Cecconi F, et al. Molecular definitions of autophagy and related processes. The EMBO Journal 2017; 36 (13):1811-36.

22. Deretic V. Autophagy in immunity and cell autonomous defense against intracellular microbes. Immunol Rev. 2011;240:92–104.

23. Münz C. Autophagy Beyond Intracellular MHC Class II Antigen Presentation. Trends Immunol. 2016;37(11):755-63.

24. Prentice E, Jerome WG, Yoshimori T, Mizushima N, Denison MR. Coronavirus replication complex formation utilizes components of cellular autophagy. J Biol Chem. 2004;279:10136-41.

25. Ulasli M, Verheije MH, de Haan CAM, Reggiori F. Qualitative and quantitative ultrastructural analysis of the membrane rearrangements induced by coronavirus. Cellular Microbiol. 2010;12(6):doi.org/10.1111/j.462-5822.2010.01437.x.

26. Miao G, Zhao H, Li Y, Ji M, Chen Y, Shi Y, et al. ORF3a of the COVID-19 virus SARS-CoV-2 blocks HOPS complex-mediated assembly of the SNARE complex required for autolysosome formation. Developmental Cell. 2021;56:427–42.

27. Yuan Z, Hu B, Xiao H, Tan X, Li Y, Tang K, et al. The E3 Ubiquitin Ligase RNF5 Facilitates SARS-CoV-2 Membrane Protein-Mediated Virion Release. mBio. 2022;13(1):e03168-21.

28. Dupont N, Chauhan S, Arko-Mensah J, Castillo EF, Masedunskas A, Weigert R, et al. Neutral lipid stores and lipase PNPLA5 contribute to autophagosome biogenesis. Curr Biol. 2014;24:609–20.

29. Shpilka T, Welter E, Borovsky N, Amar N, Mari M, Reggiori F, et al. Lipid droplets and their component triglycerides and steryl esters regulate autophagosome biogenesis. EMBO J. 2015;34:2117–31.

30. Heaton NS, Randall G. Dengue virus-induced autophagy regulates lipid metabolism. Cell Host Microbe. 2010;8:422–32.

31. Zhang J, Lan Y, Li MY, Lamers MM, Fusade-Boyer M, Klemm E, et al. Flaviviruses exploit the lipid droplet protein AUP1 to trigger lipophagy and drive virus production. Cell Host Microbe. 2018;23:819–31.
