# Supplementary figures and images for: Evidence of SARS-CoV-2 infection in postmortem lung, kidney, and liver samples, revealing cellular targets involved in COVID-19 pathogenesis
Source: Arch Virol. 2023 Feb 26;168(3):96. doi: 10.1007/s00705-023-05711-y (PMC9968404; doi:10.1007/s00705-023-05711-y)

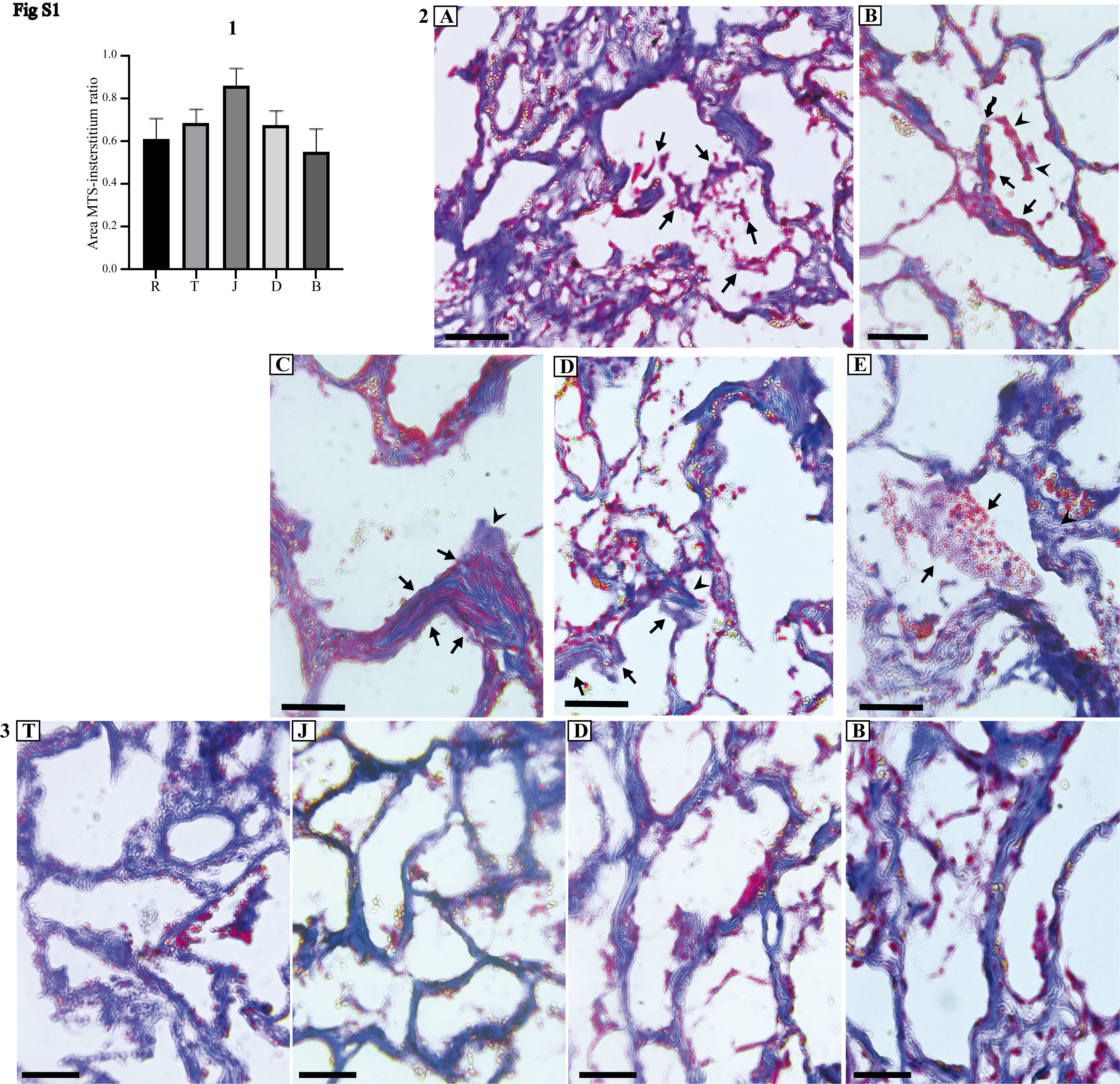

Supplement: Supplementary file 2 — Supplementary file2 (TIF 20487 KB) [file 705_2023_5711_MOESM2_ESM.tif]

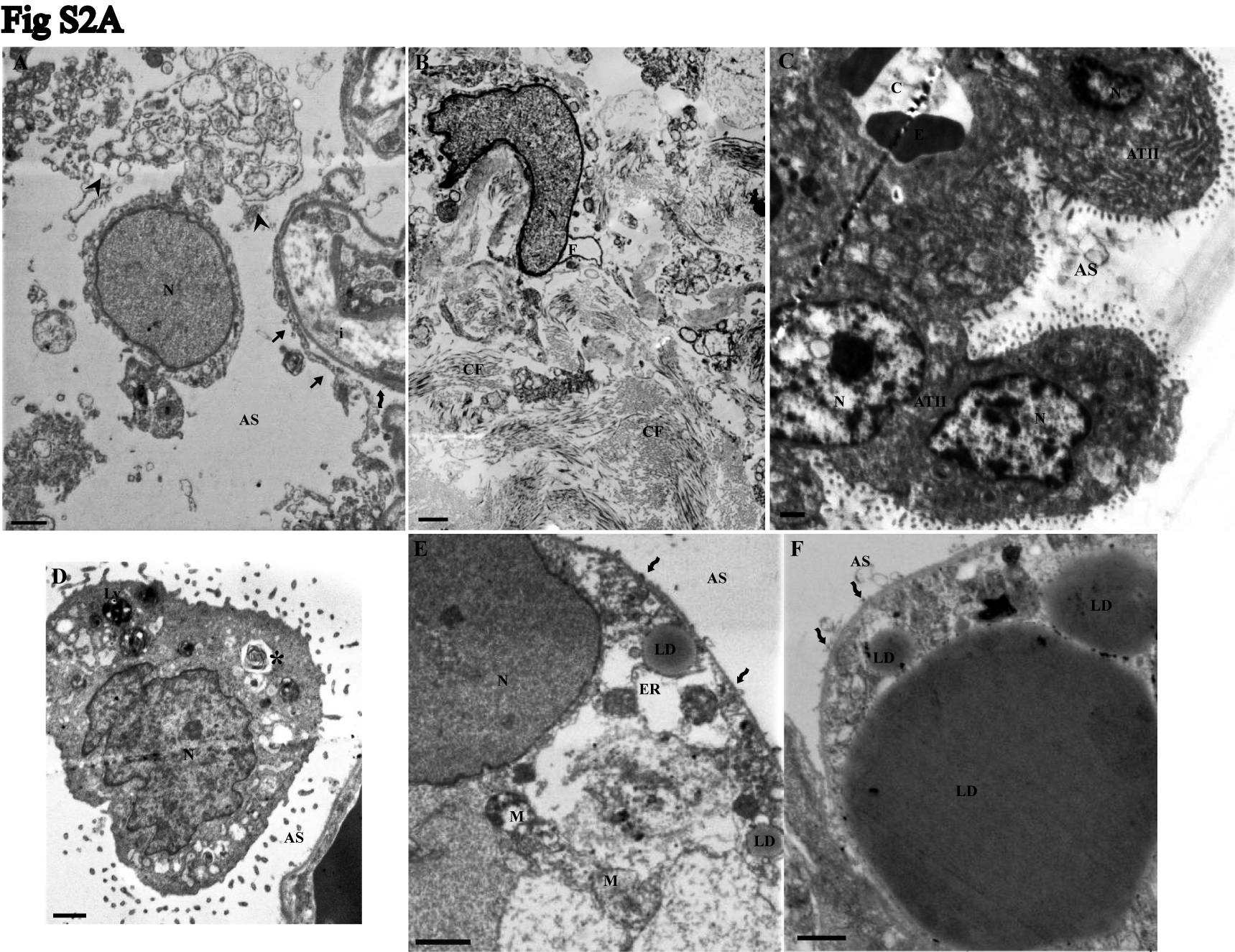

Supplement: Supplementary file 3 — Supplementary file3 (TIF 8936 KB) [file 705_2023_5711_MOESM3_ESM.tif]

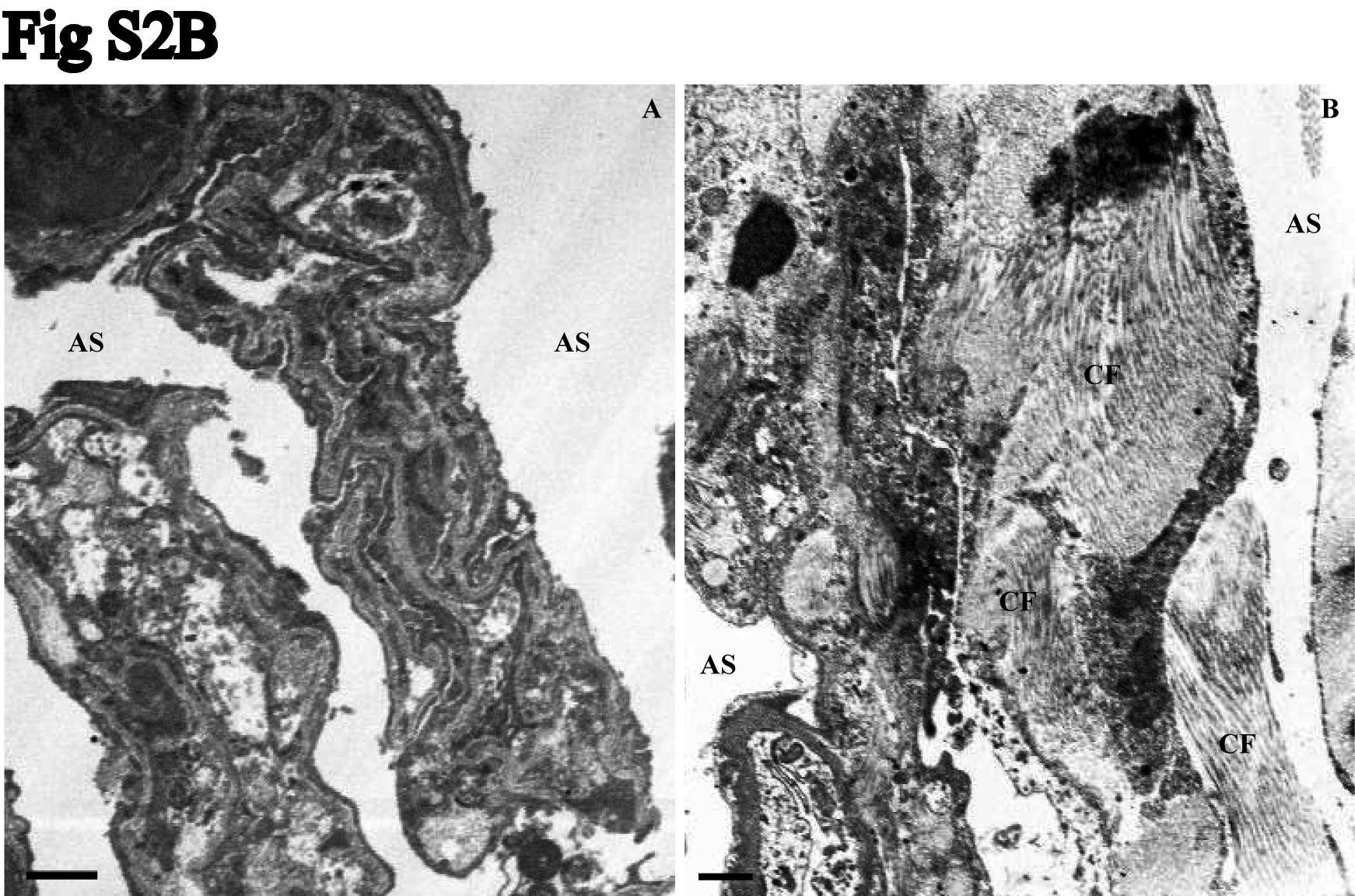

Supplement: Supplementary file 4 — Supplementary file4 (TIF 7876 KB) [file 705_2023_5711_MOESM4_ESM.tif]

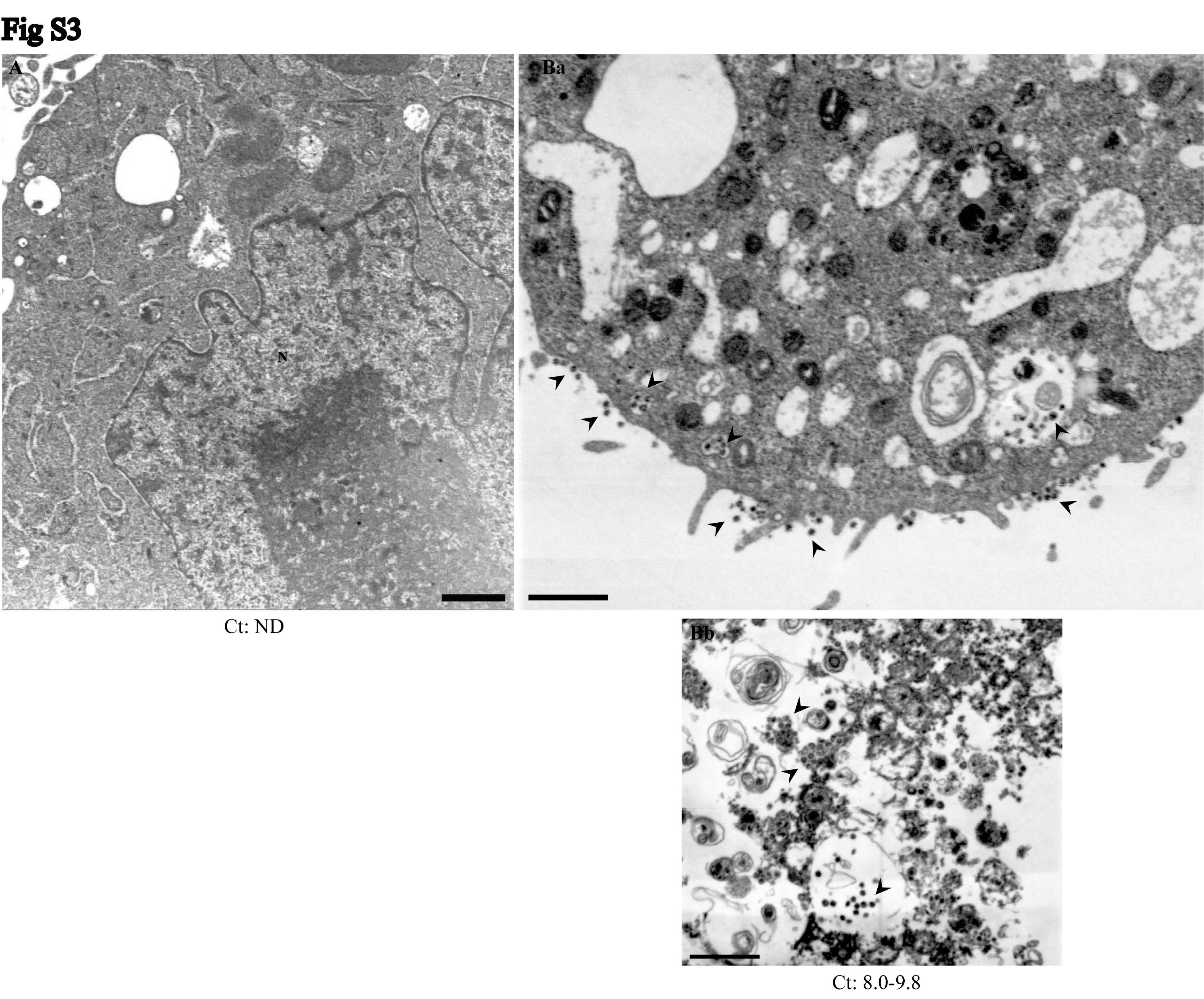

Supplement: Supplementary file 5 — Supplementary file5 (TIF 8229 KB) [file 705_2023_5711_MOESM5_ESM.tif]

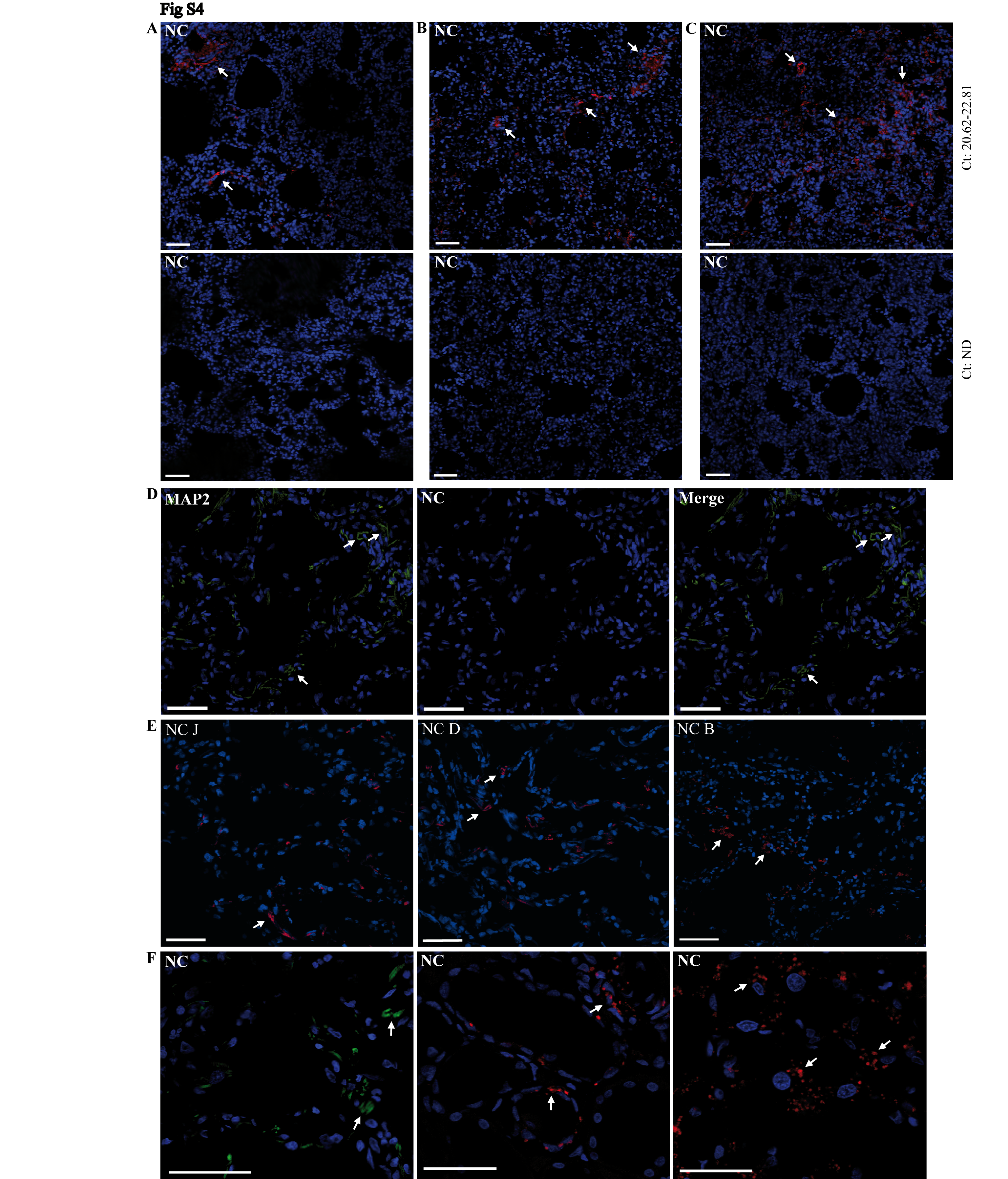

Supplement: Supplementary file 6 — Supplementary file6 (TIF 6929 KB) [file 705_2023_5711_MOESM6_ESM.tif]

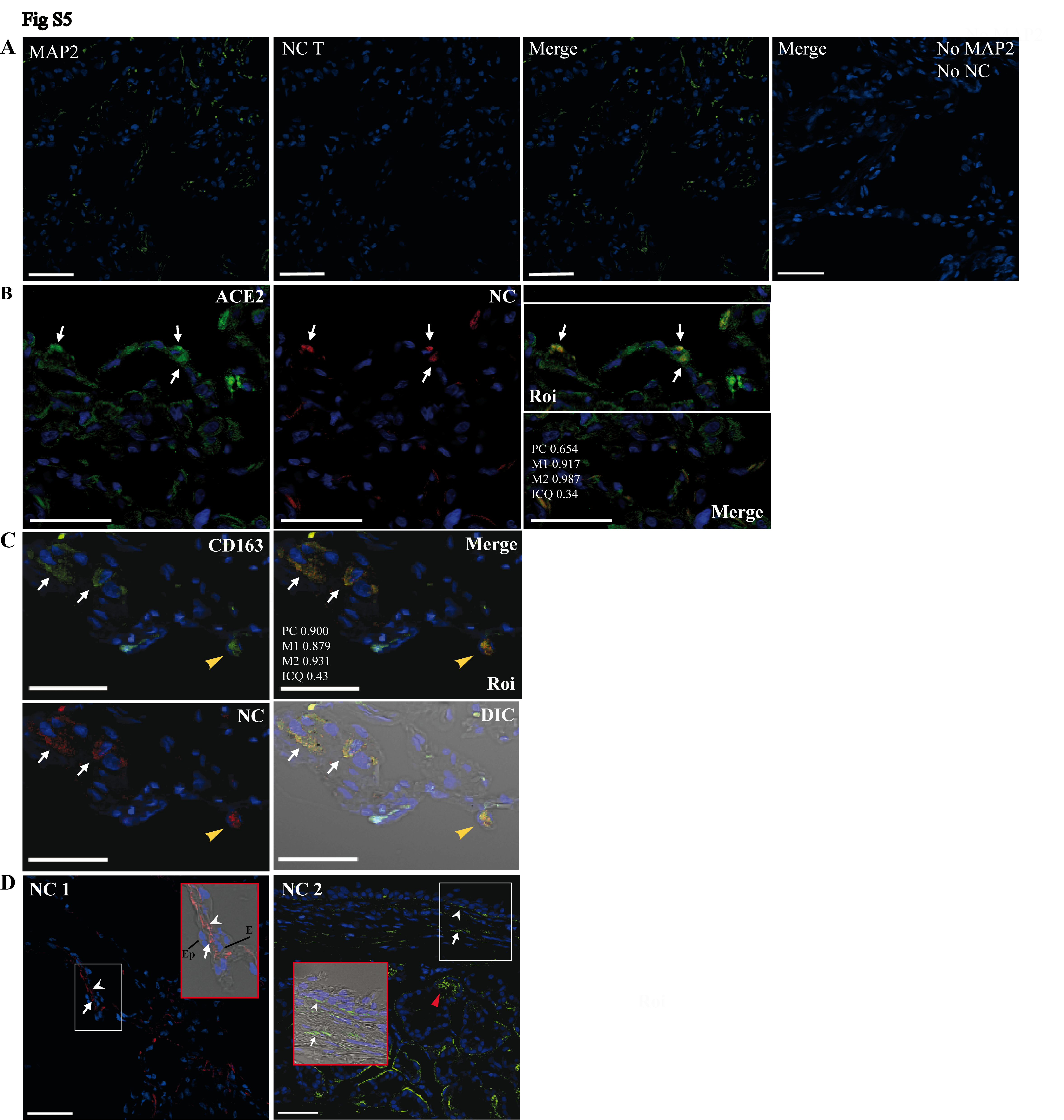

Supplement: Supplementary file 7 — Supplementary file7 (TIF 7671 KB) [file 705_2023_5711_MOESM7_ESM.tif]

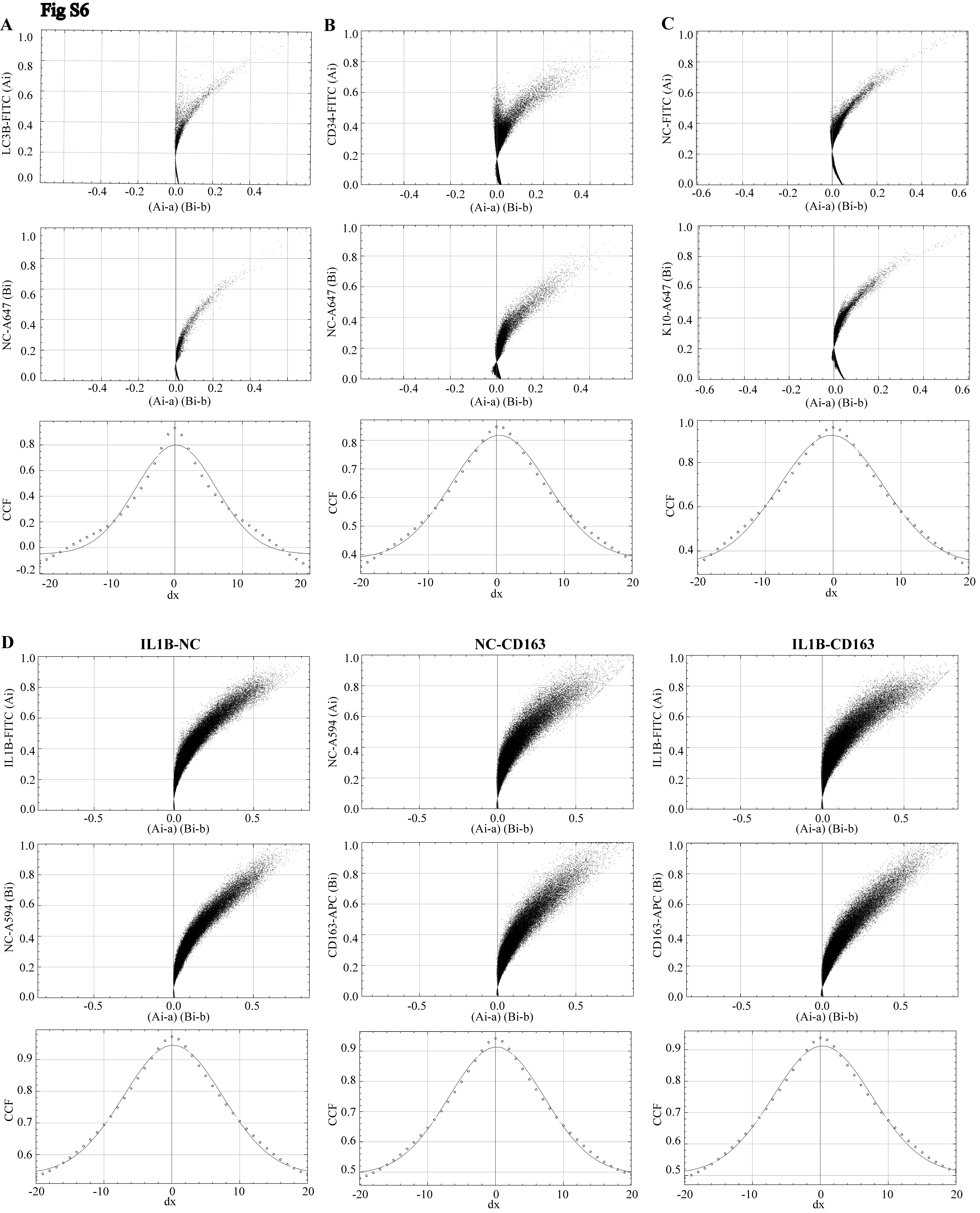

Supplement: Supplementary file 8 — Supplementary file8 (TIF 1263 KB) [file 705_2023_5711_MOESM8_ESM.tif]

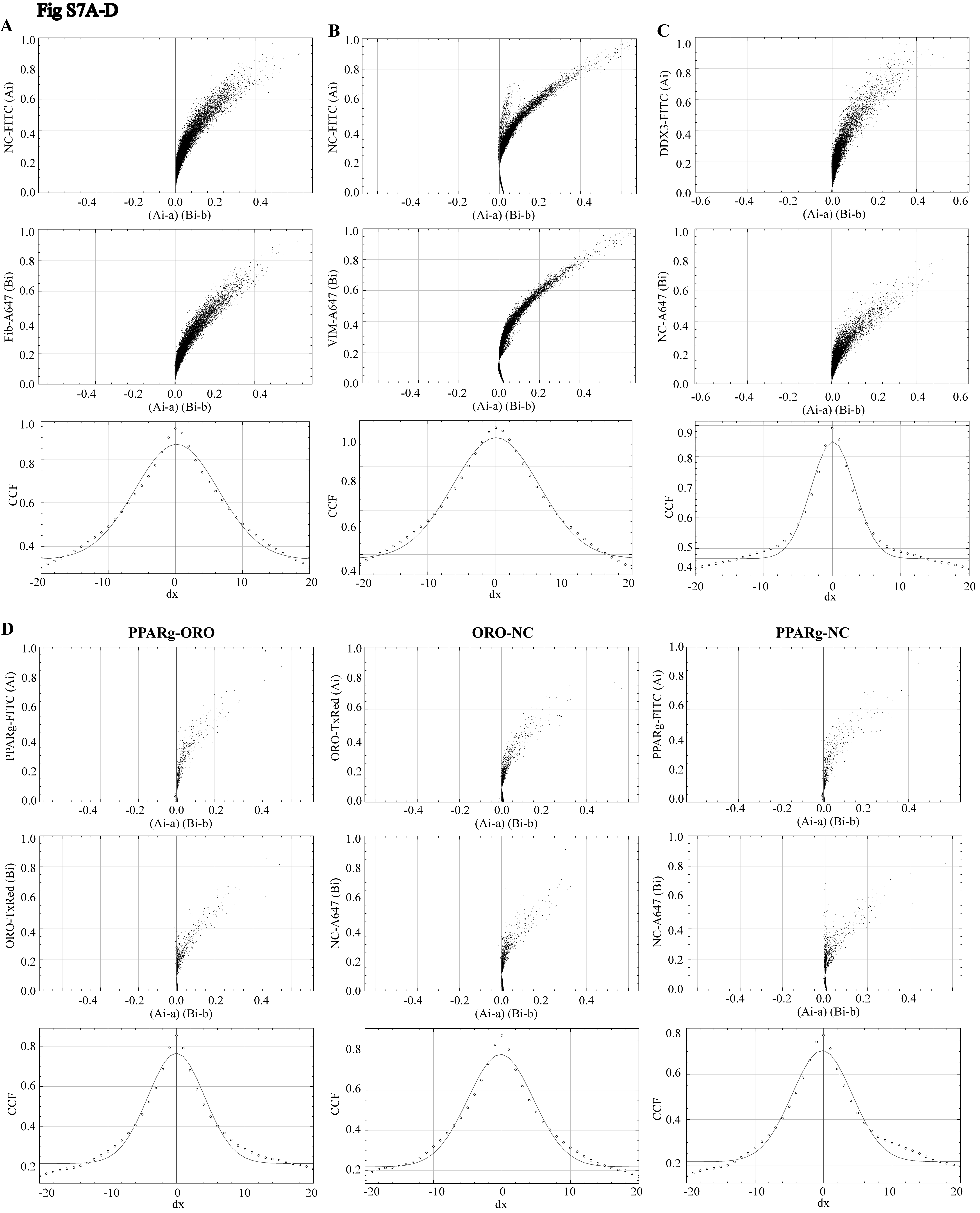

Supplement: Supplementary file 9 — Supplementary file9 (TIF 1165 KB) [file 705_2023_5711_MOESM9_ESM.tif]

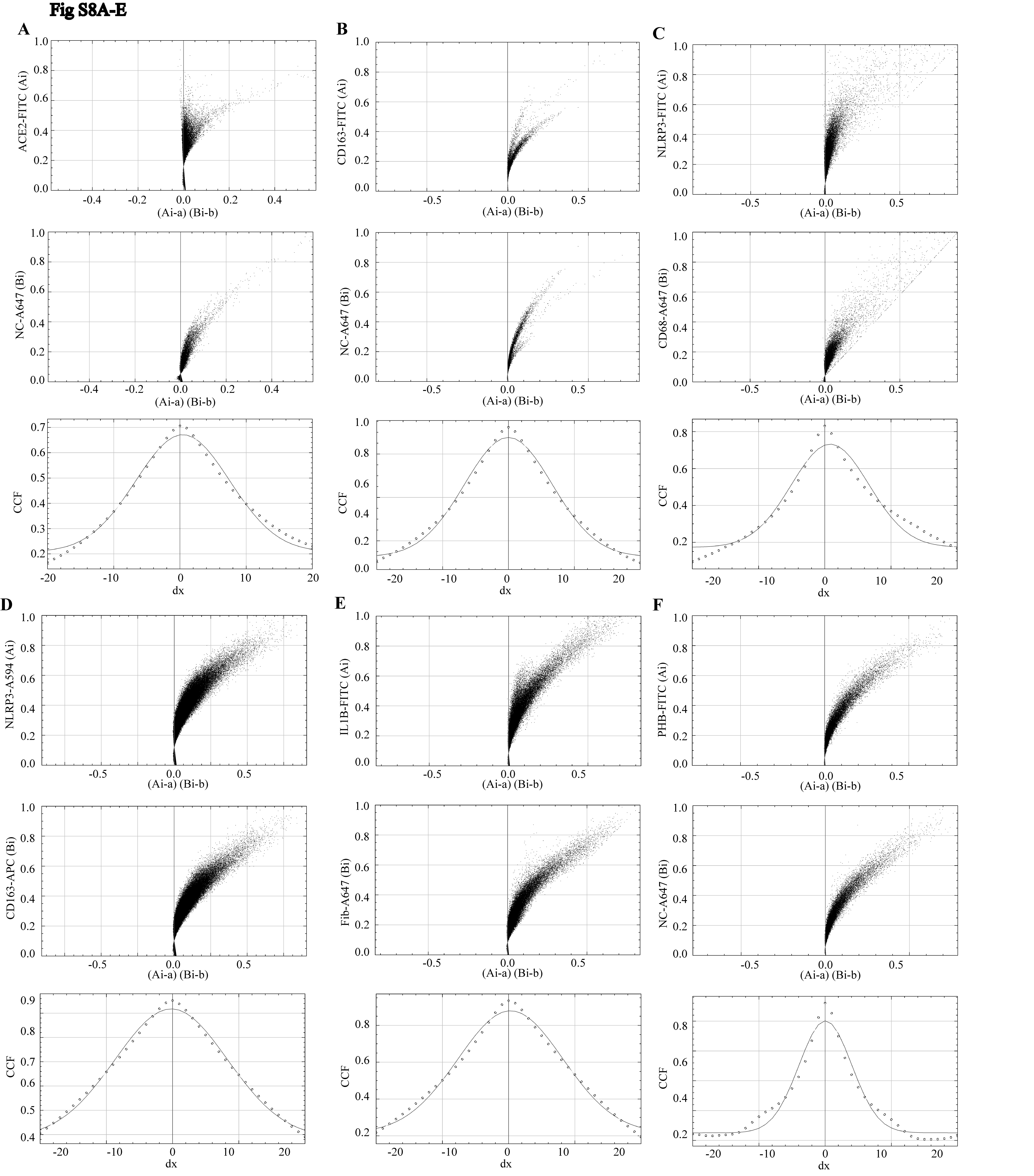

Supplement: Supplementary file 10 — Supplementary file10 (TIF 1223 KB) [file 705_2023_5711_MOESM10_ESM.tif]

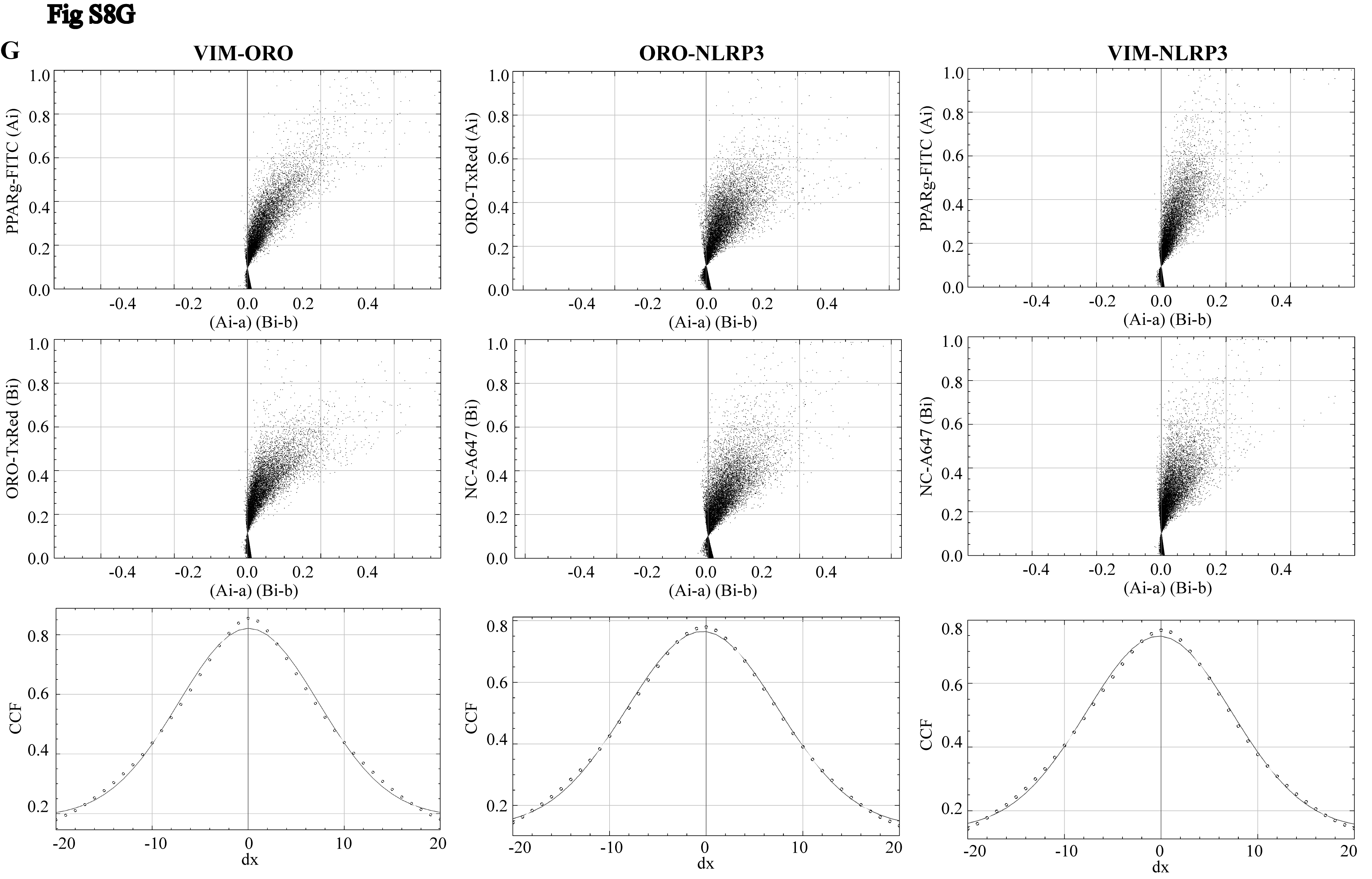

Supplement: Supplementary file 11 — Supplementary file11 (TIF 644 KB) [file 705_2023_5711_MOESM11_ESM.tif]

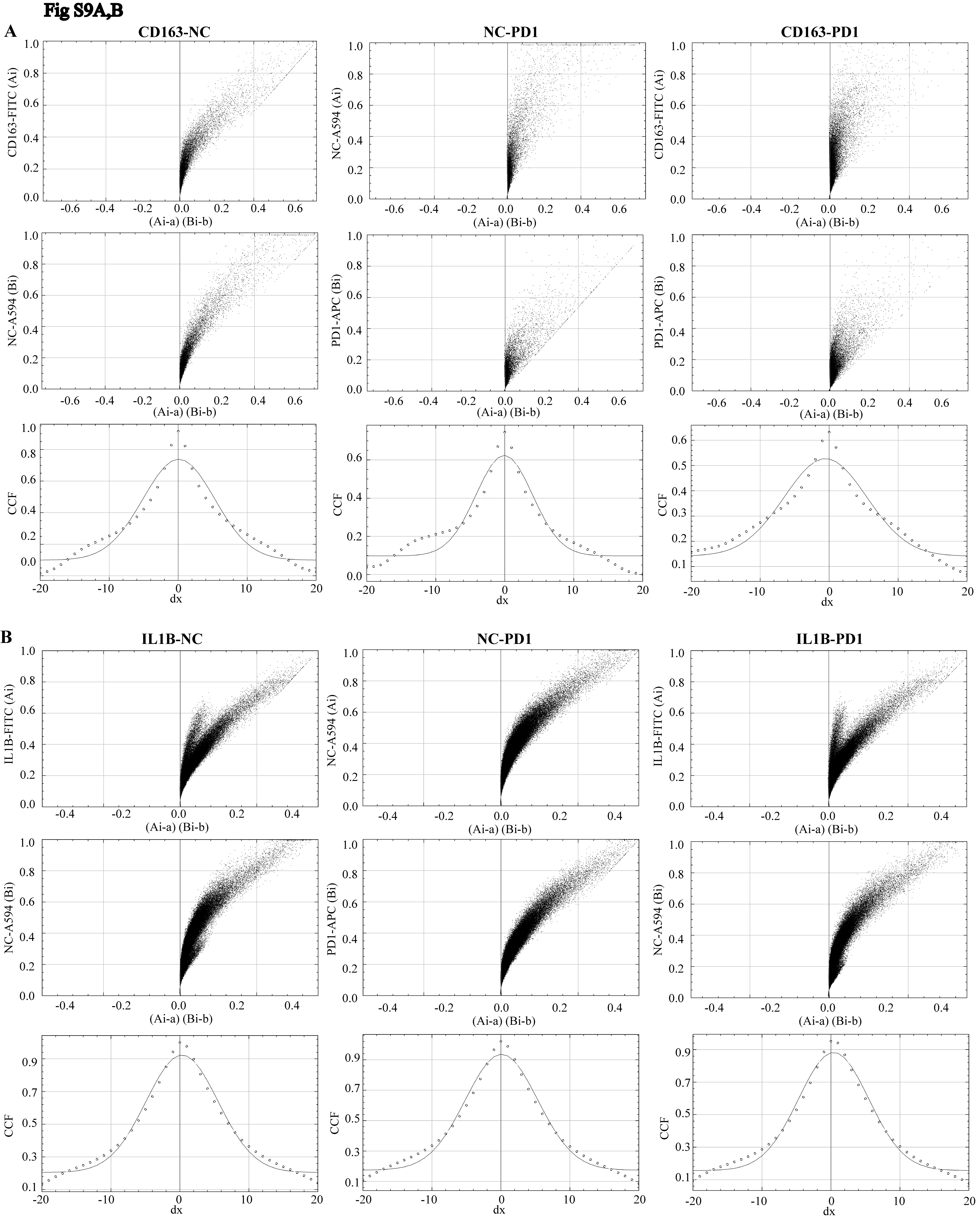

Supplement: Supplementary file 12 — Supplementary file12 (TIF 1235 KB) [file 705_2023_5711_MOESM12_ESM.tif]

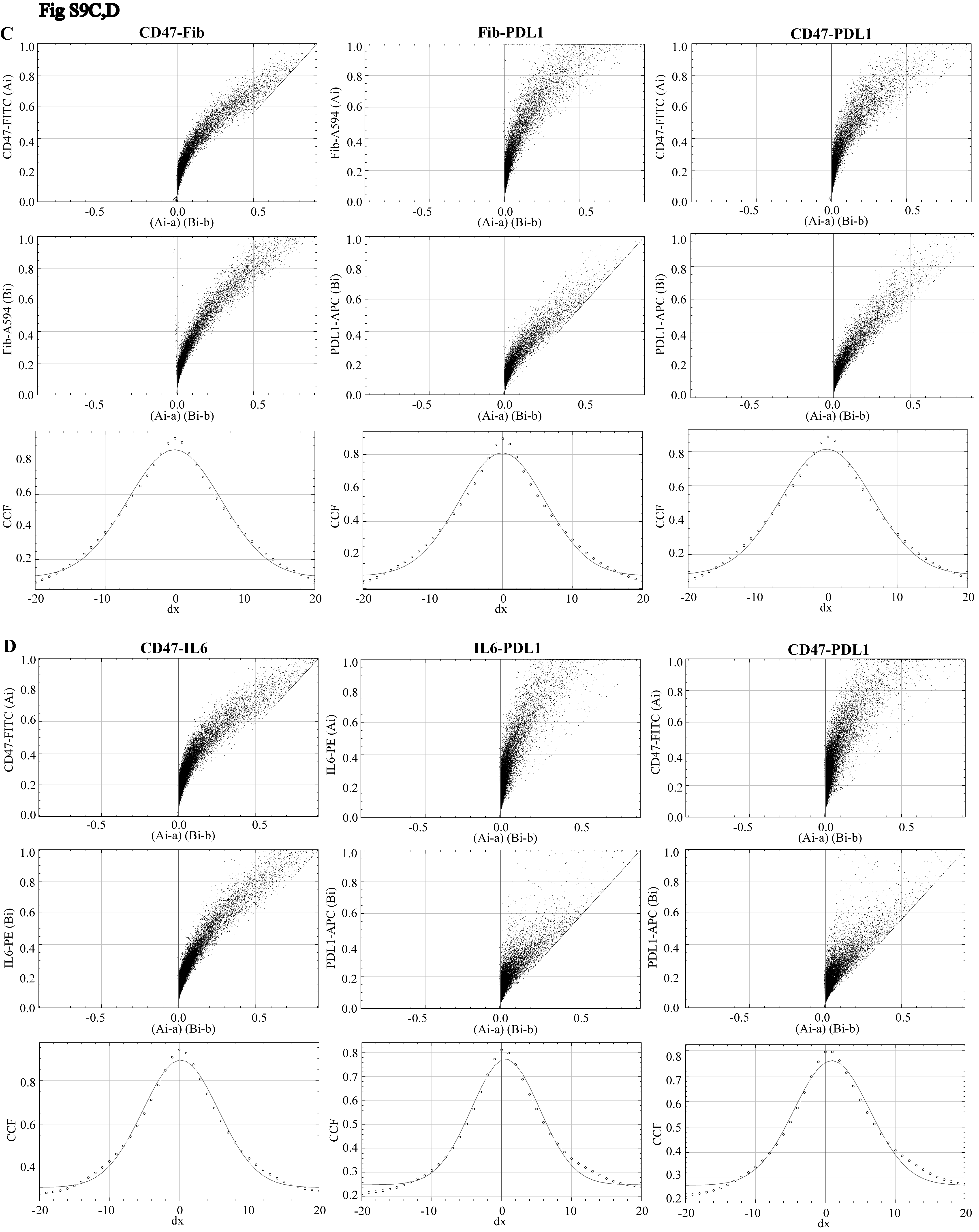

Supplement: Supplementary file 13 — Supplementary file13 (TIF 1254 KB) [file 705_2023_5711_MOESM13_ESM.tif]

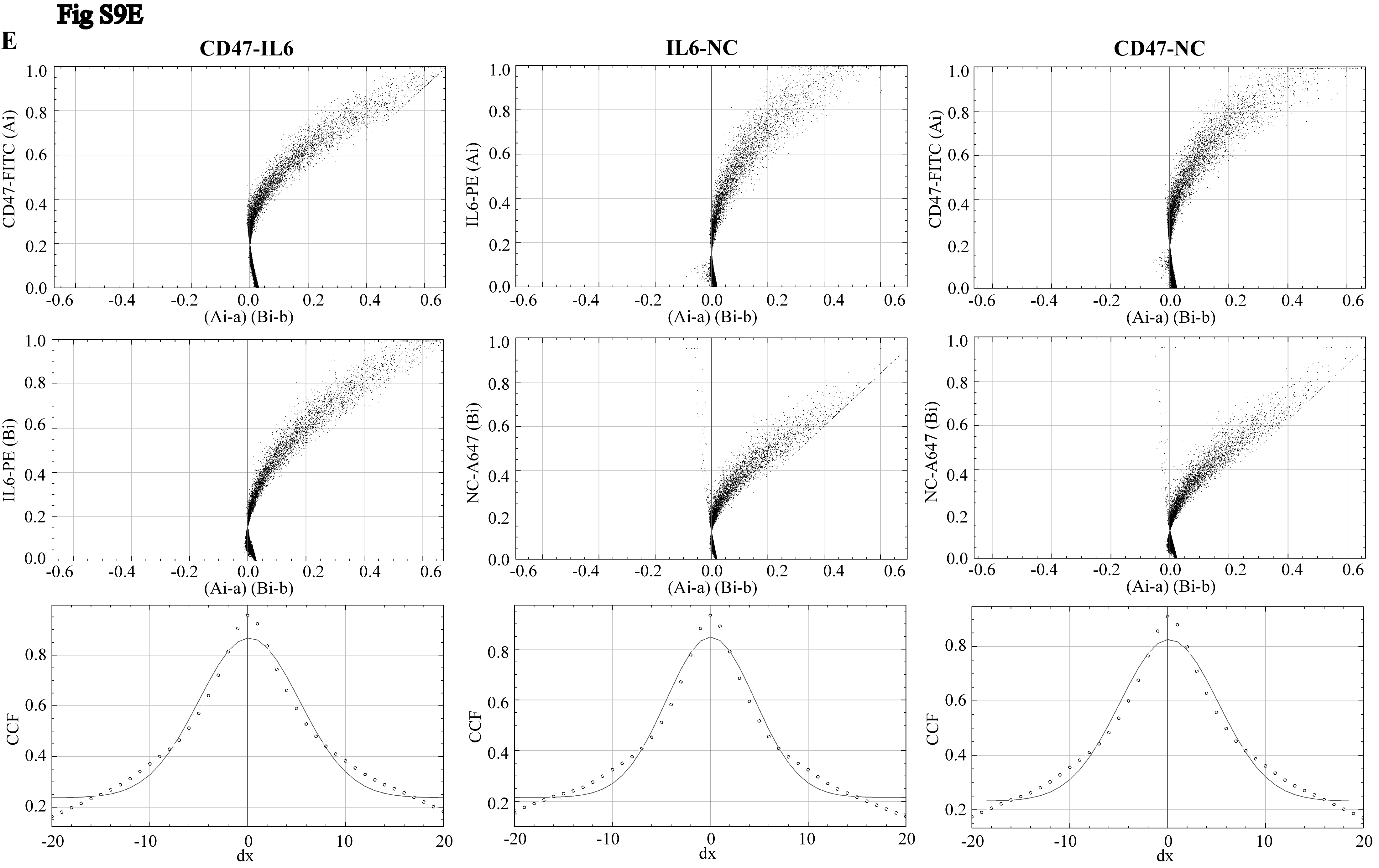

Supplement: Supplementary file 14 — Supplementary file14 (TIF 642 KB) [file 705_2023_5711_MOESM14_ESM.tif]

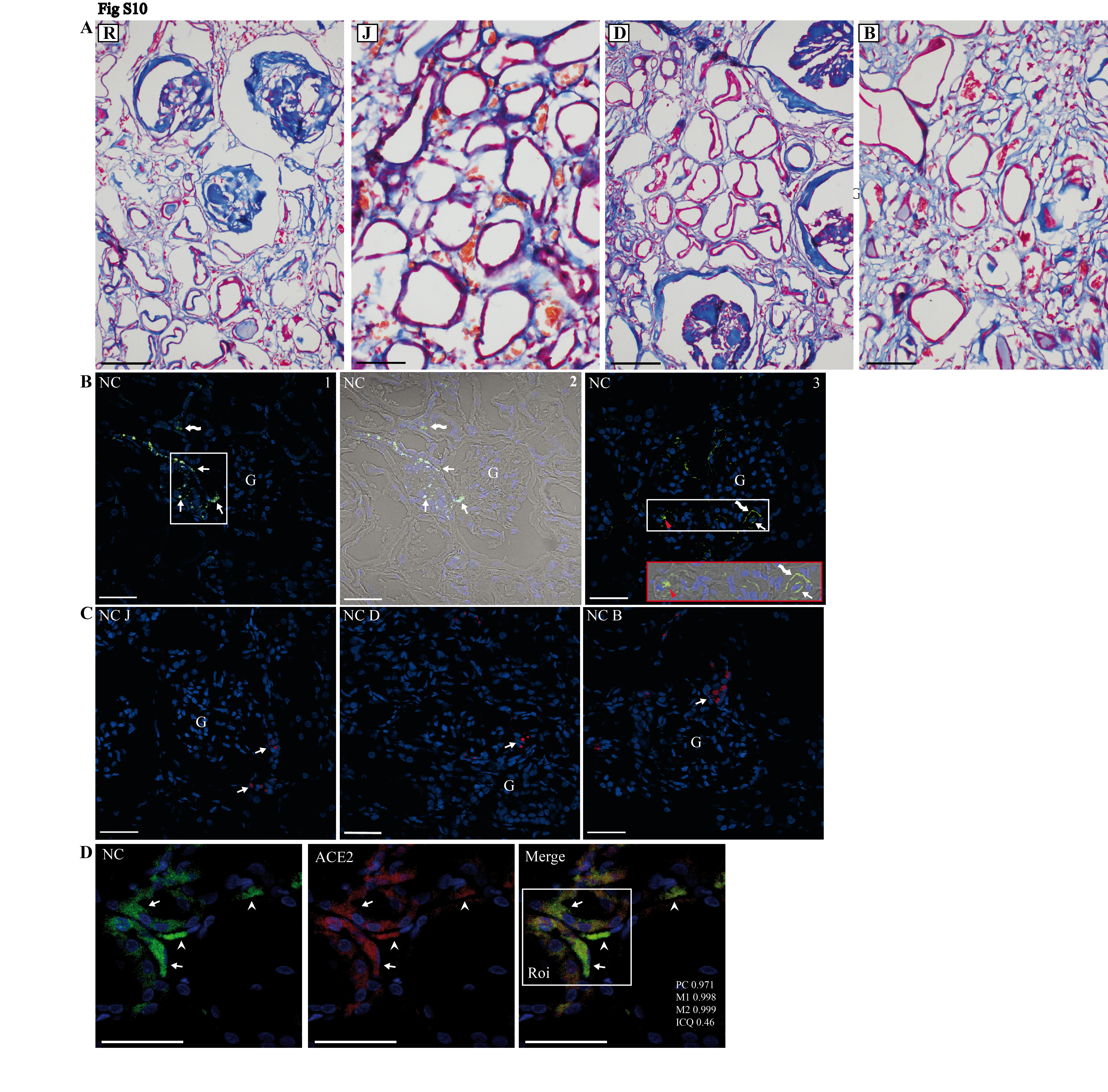

Supplement: Supplementary file 15 — Supplementary file15 (JPG 6770 KB) [file 705_2023_5711_MOESM15_ESM.jpg]

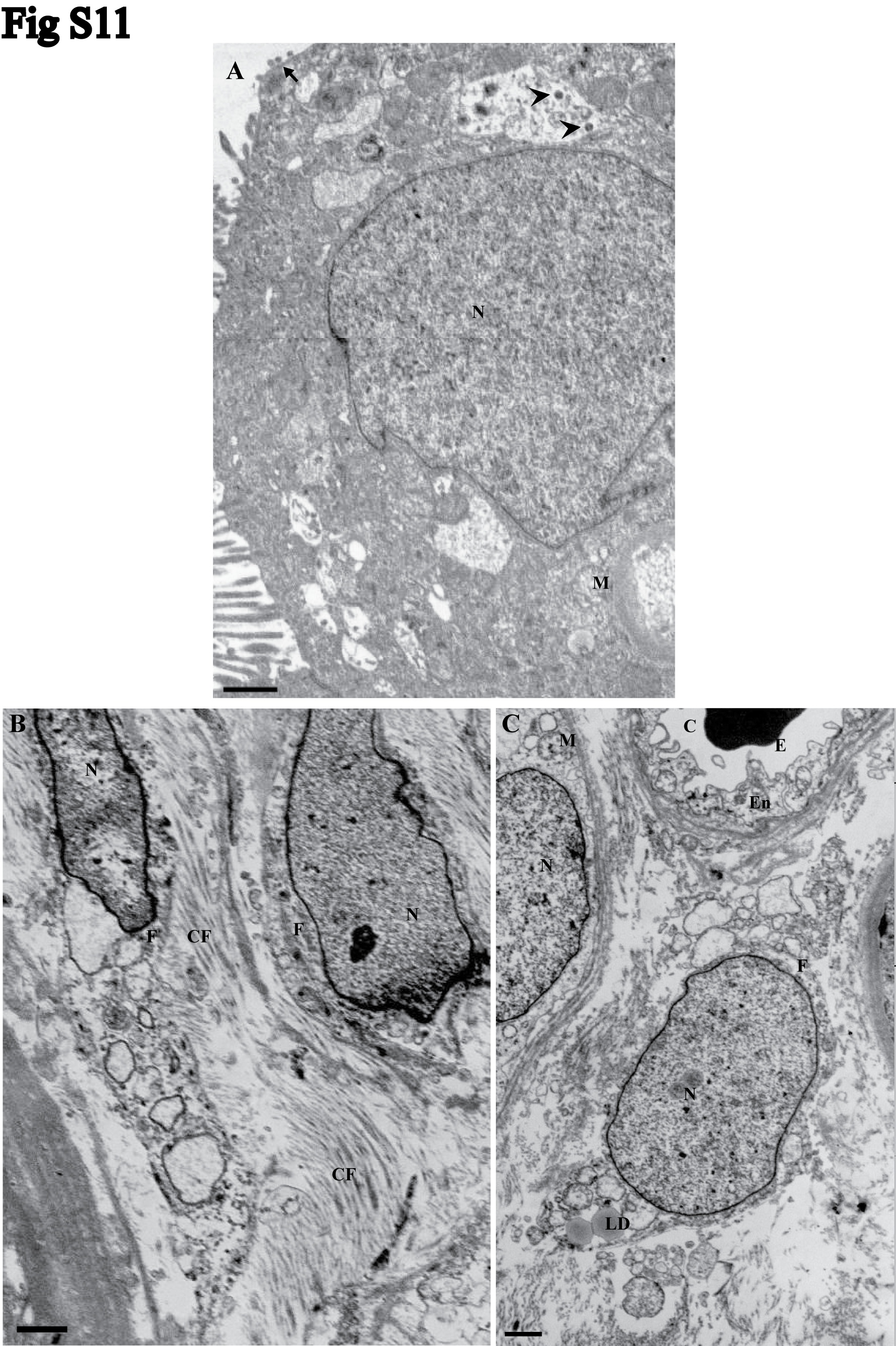

Supplement: Supplementary file 16 — Supplementary file16 (TIF 6423 KB) [file 705_2023_5711_MOESM16_ESM.tif]

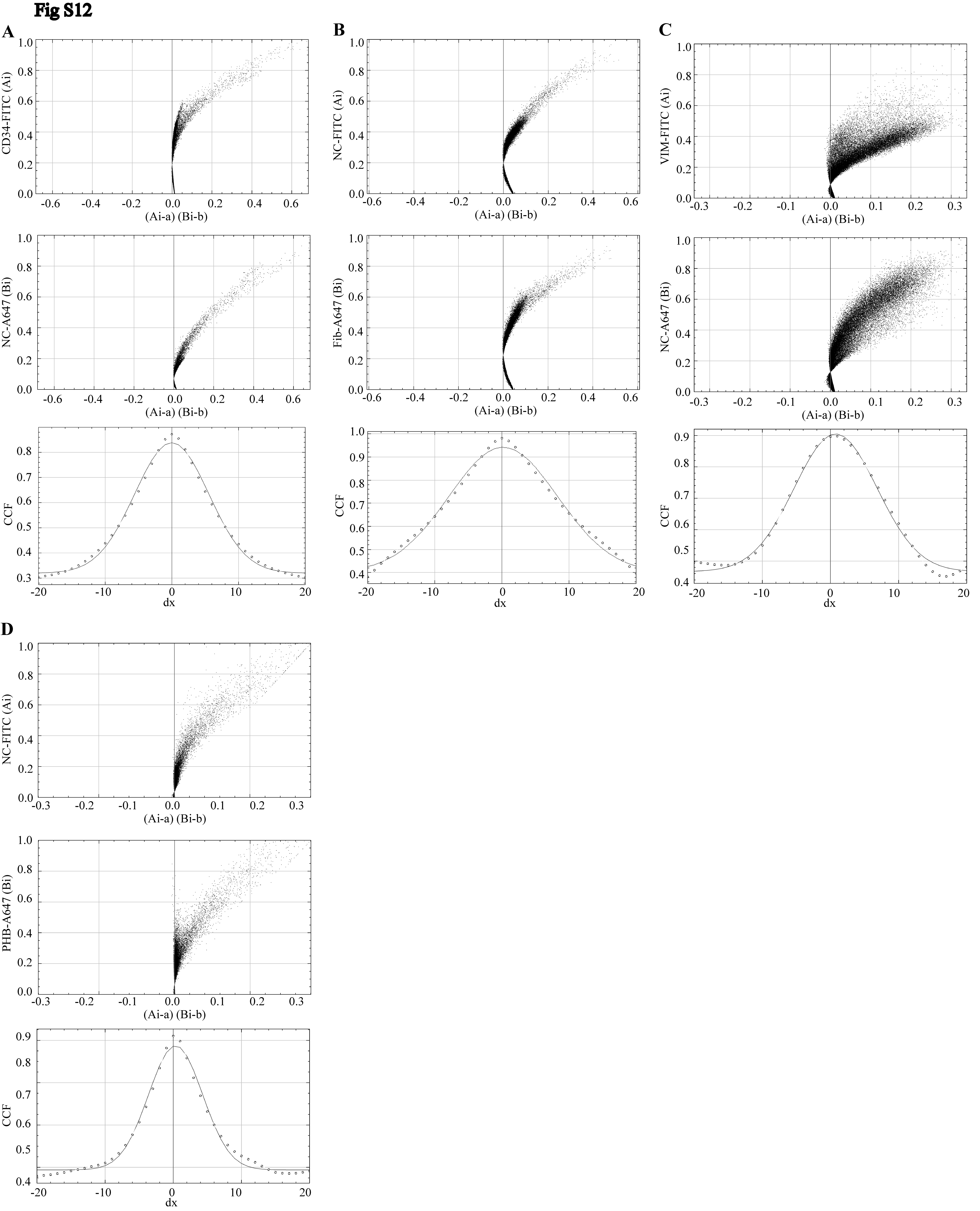

Supplement: Supplementary file 17 — Supplementary file17 (TIF 1083 KB) [file 705_2023_5711_MOESM17_ESM.tif]

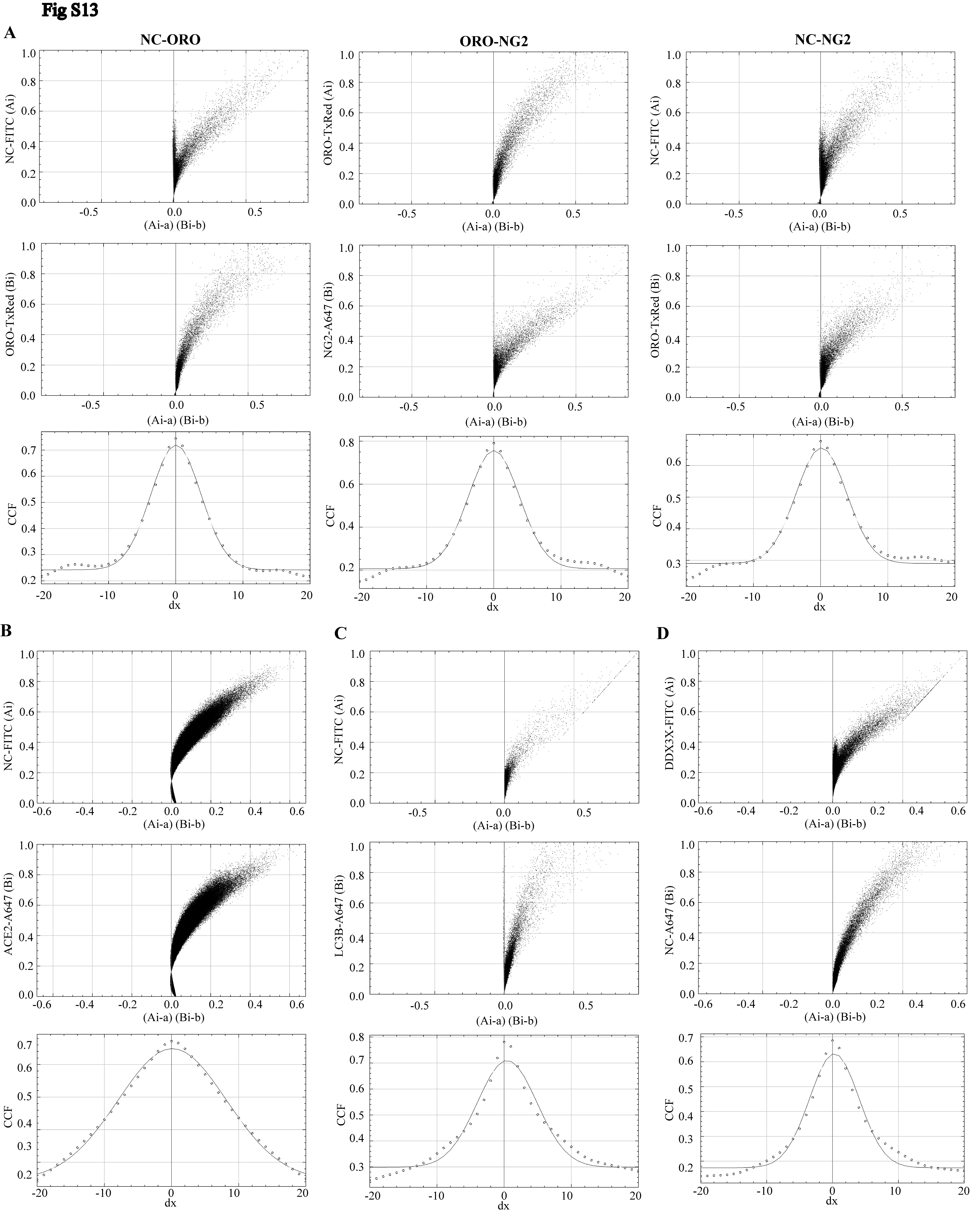

Supplement: Supplementary file 18 — Supplementary file18 (TIF 1226 KB) [file 705_2023_5711_MOESM18_ESM.tif]

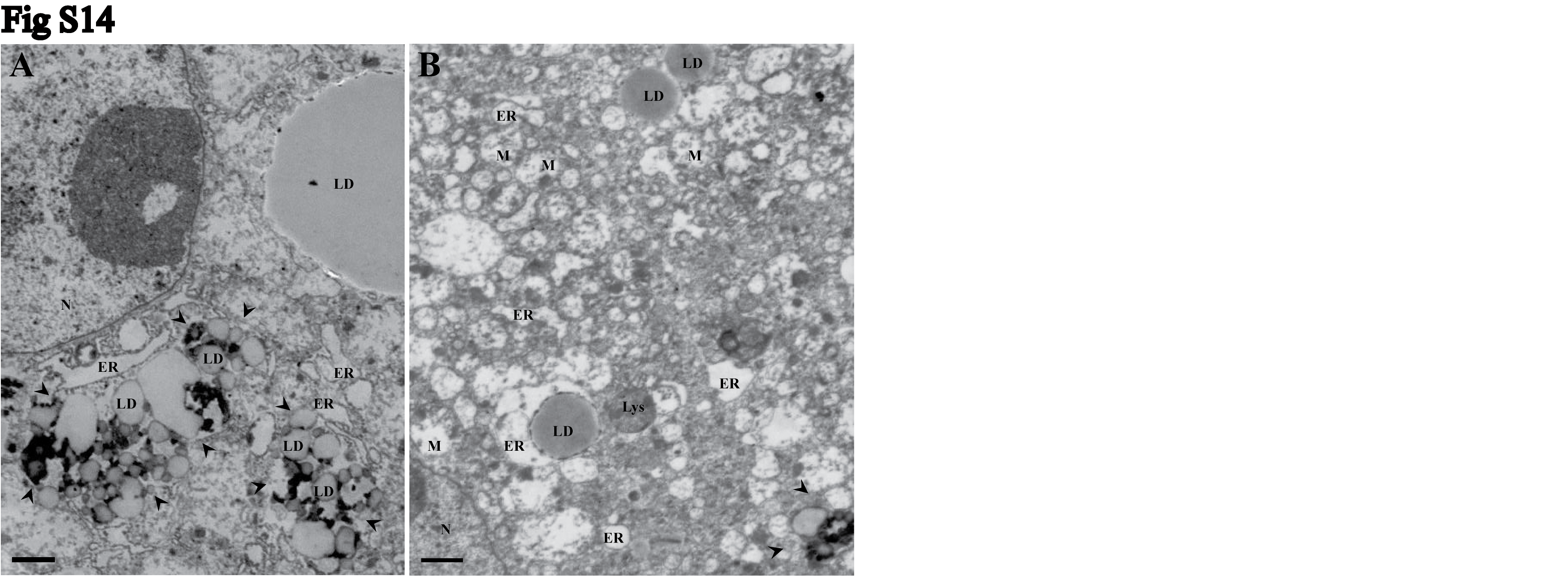

Supplement: Supplementary file 19 — Supplementary file19 (TIF 3955 KB) [file 705_2023_5711_MOESM19_ESM.tif]

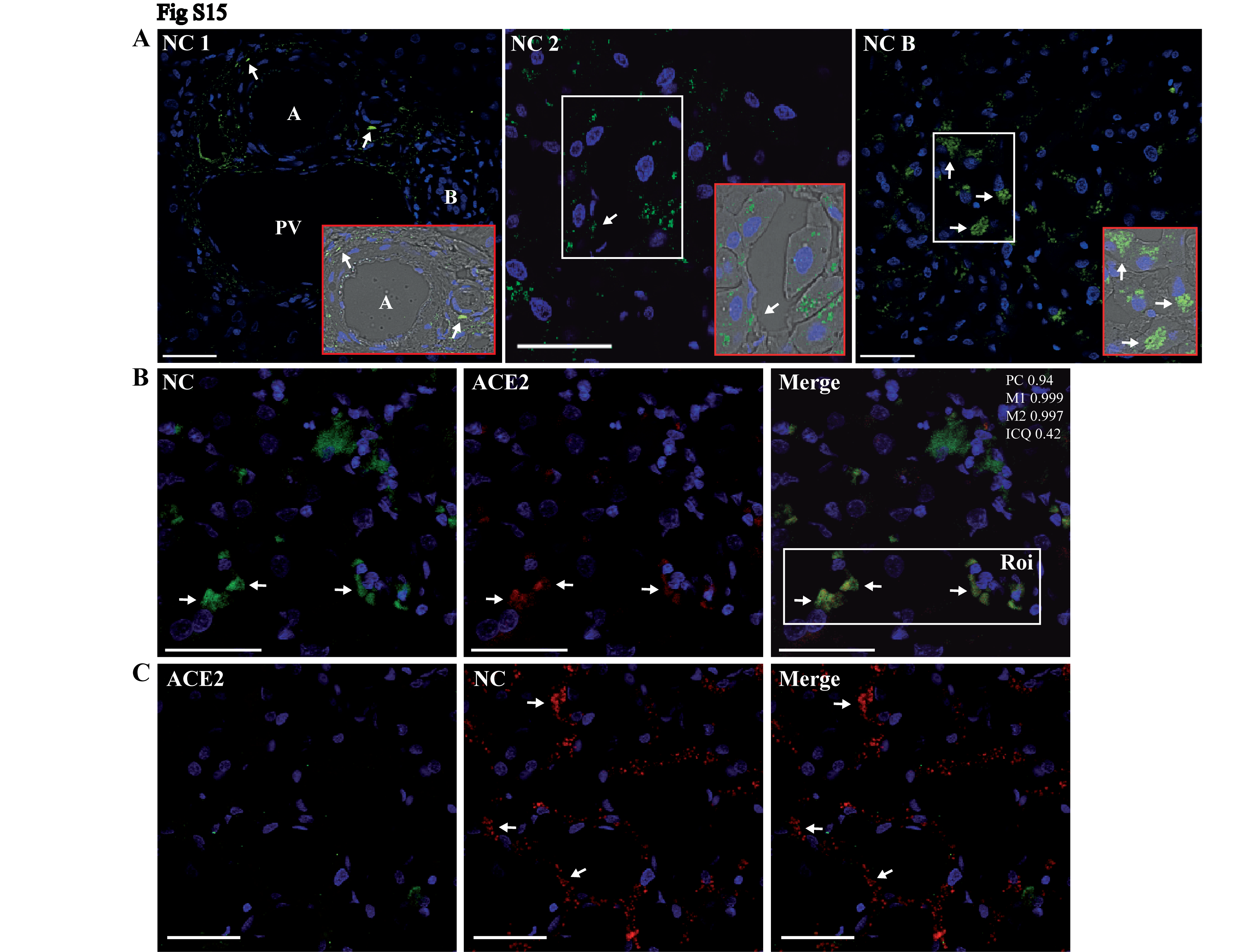

Supplement: Supplementary file 20 — Supplementary file20 (TIF 7538 KB) [file 705_2023_5711_MOESM20_ESM.tif]

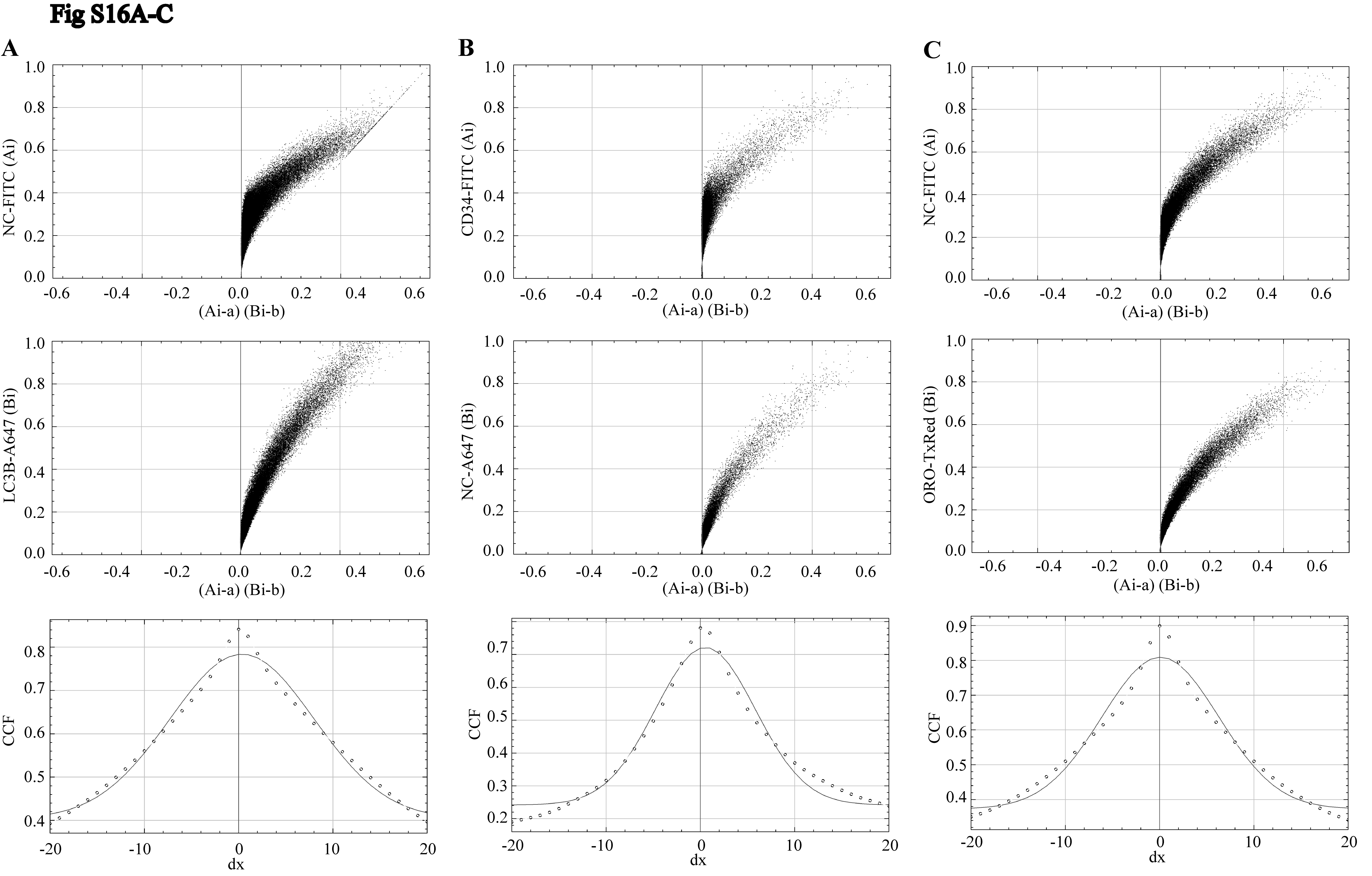

Supplement: Supplementary file 21 — Supplementary file21 (TIF 639 KB) [file 705_2023_5711_MOESM21_ESM.tif]

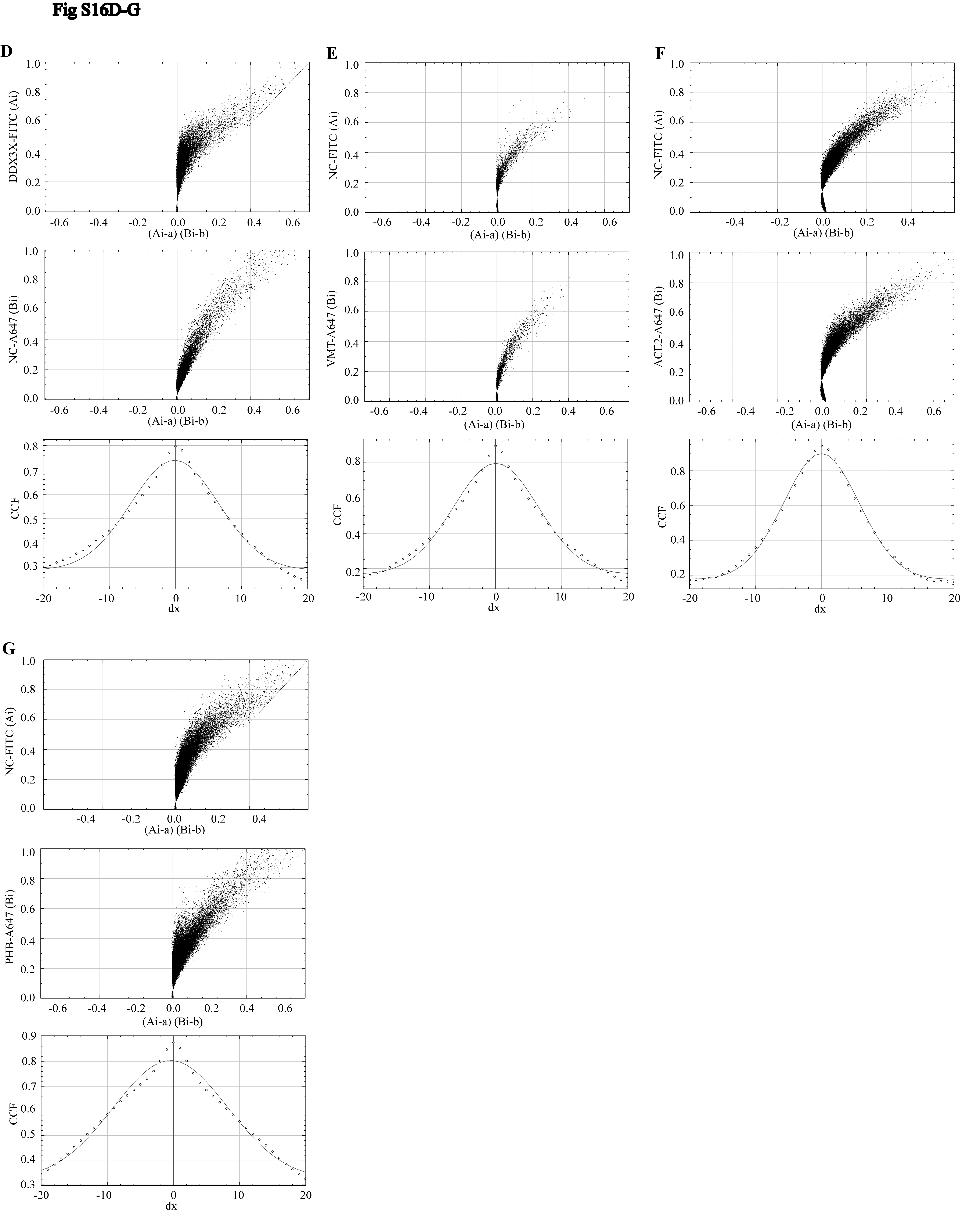

Supplement: Supplementary file 22 — Supplementary file22 (TIF 1114 KB) [file 705_2023_5711_MOESM22_ESM.tif]

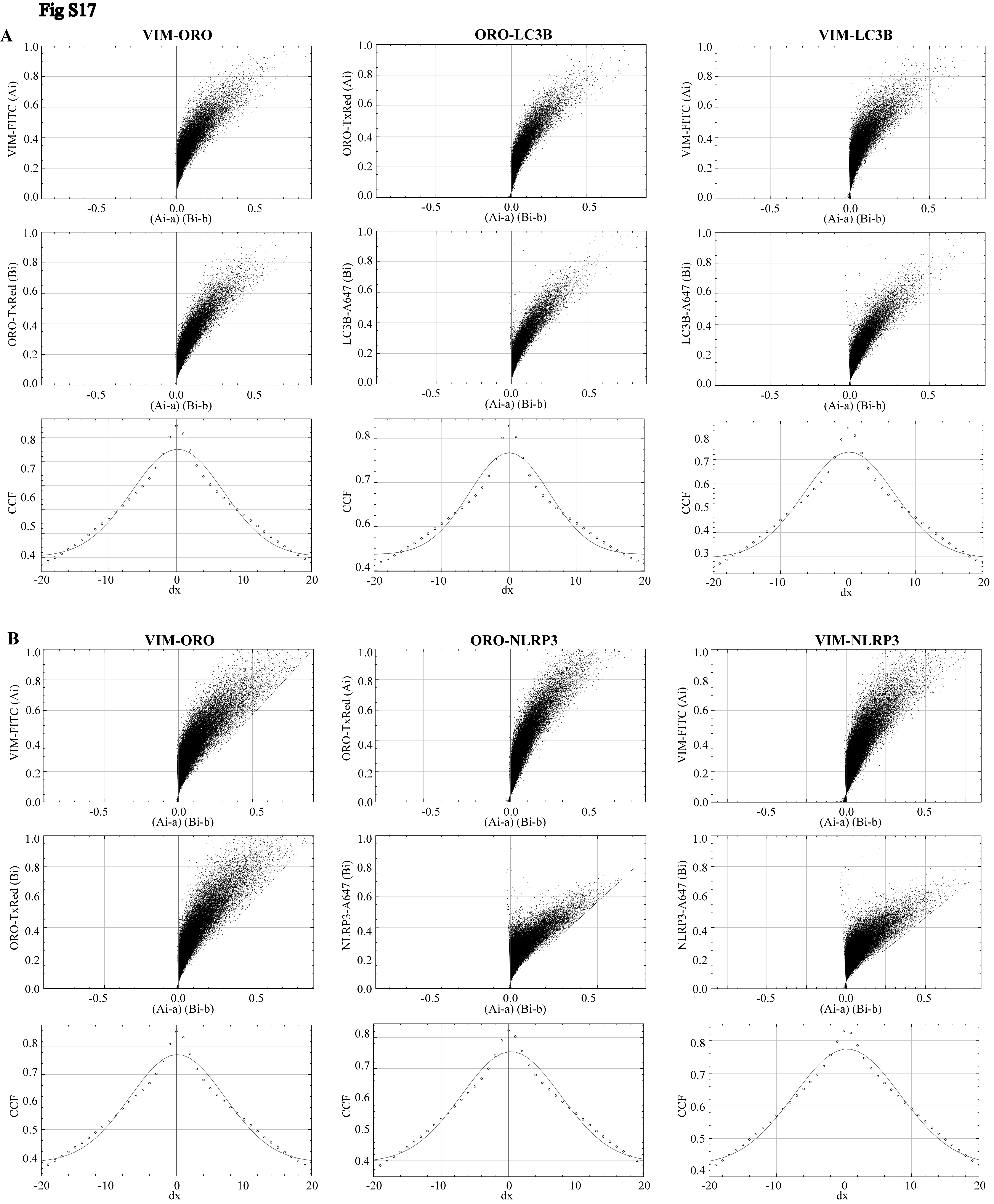

Supplement: Supplementary file 23 — Supplementary file23 (TIF 1338 KB) [file 705_2023_5711_MOESM23_ESM.tif]
